# A Validated Multiplex Real-Time PCR Assay for the Diagnosis of Infectious *Leptospira* spp.: A Novel Assay for the Detection and Differentiation of Strains From Both Pathogenic Groups I and II

**DOI:** 10.3389/fmicb.2020.00457

**Published:** 2020-03-20

**Authors:** Lester J. Pérez, Saraswathi Lanka, Vanessa J. DeShambo, Richard L. Fredrickson, Carol W. Maddox

**Affiliations:** ^1^Veterinary Diagnostic Laboratory, College of Veterinary Medicine, University of Illinois at Urbana–Champaign, Urbana, IL, United States; ^2^Department of Clinical Veterinary Medicine, College of Veterinary Medicine, University of Illinois at Urbana–Champaign, Urbana, IL, United States; ^3^Department of Pathobiology, College of Veterinary Medicine, University of Illinois at Urbana–Champaign, Urbana, IL, United States

**Keywords:** *Leptospira* spp., multiplex real-time strategy, pathogenic group I and II, validation, molecular diagnostic

## Abstract

Leptospirosis is recognized as the most globally widespread reemerging zoonosis and represents a serious threat for both human and animal health. Indeed, leptospirosis is linked to more than 60,000 human deaths per year and to incalculable economic burden as consequence of medical treatment costs and livestock loss. The increasing number of reports from species of pathogenic *Leptospira* spp. group II causing disease in both humans and animals constitutes an additional concern to the complex epidemiology of this zoonotic agent. Diagnostic methods based on qPCR have improved the diagnosis of *Leptospira* spp. in terms of cost, time, and reliability, but most of the validated assays fail to detect species from the pathogenic group II. Hence, the current study was aimed to develop and validate a novel multiplex qPCR to enable the specific and selective detection of the whole group of infectious *Leptospira* spp., including both pathogenic groups I and II and moreover, selectively discriminate between them. To fit the “fitness of purpose” for the specific detection of infectious *Leptospira* spp. and further discrimination between both pathogenic groups three target regions on the *16S RNA* gene were selected. These targets facilitated a broad and selective spectrum for the detection of all infectious *Leptospira* spp. with the exclusion of all saprophytic groups and the novel clade of environmental *Leptospira* spp. The analytical sensitivity (ASe) showed by the new assay also enables a wide window of detection for the agent at different stages of infection since the assay was able to efficiently detect at 95% of confidence ∼5 leptospires/reaction. From the evaluation of the analytical specificity (ASp) by *in silico* and *in vitro* approaches, it was congruently revealed that the primers and probes selected only recognized the specific targets for which the assay was intended. Bayesian latent class analysis of performance of the new assay on 684 clinical samples showed values of diagnostic sensitivity of 99.8% and diagnostic specificity of 100%. Thus, from the evaluation of the analytical and diagnostic parameters, the new multiplex qPCR assay is a reliable method for the diagnosis of *Leptospira* spp.

## Introduction

Leptospirosis is globally recognized as the most widespread reemerging zoonosis (Adler, 2015). This severe infectious disease has an estimated incidence of 1.03 million cases in humans every year, with approximately 59,000 deaths (Costa et al., 2015; Torgerson et al., 2015). Leptospirosis is also causing major health problems in many animal species including: dogs, cattle, swine, horses, sheep among others (Ellis, 2015). In humans, leptospirosis presents a variety of clinical manifestations, from asymptomatic subclinical infection to severe potentially fatal disease (Vk et al., 2018). Meanwhile, in animals, the disease is essentially different from human leptospirosis and it is characterized by acute clinical features, with presence of abortion and/or multiple organ injuries as principal clinical signs, but chronic infections can also occur, resulting in significant economic losses (Stobart Gallagher and Dunn, 2019). Therefore, leptospirosis is considered a global life-threatening disease in public health which can have a major impact on animal health.

Leptospirosis is caused by Gram-negative infectious spirochetes of the genus *Leptospira*, family *Leptospiraceae* (Levett and Picardeau, 2018). The initial classification of this genus only comprised two species: *Leptospira interrogans* (pathogenic strains) and *Leptospira biflexa* (saprophytic strains) (Johnson and Rogers, 1964; Johnson and Harris, 1967). From these two species, further classifications have been established based on different criteria including: similarity of surface-exposed epitopes as determined by using the cross-agglutinin absorption test (CAAT) (Fairbrother, 1984), phenotypic characteristics (Johnson et al., 1970), and DNA base composition (Ramadass et al., 1990a, b, 1992). Thus, more than 24 serogroups and 300 serovars have been recognized to date (Paiva-Cardoso et al., 2013; Valverde Mde et al., 2013). However, it is important to denote that despite the serologic classification has been widely accepted, it lacks taxonomic standing. In fact, initial studies have been conducted in order to replace this classification with a genomic approach (Levett and Picardeau, 2018).

Based on genetic analysis, the *16S* RNA gene has been found as a proper marker for classification purposes in *Leptospira* genus. The topology obtained based on the analysis of this marker clearly showed an obvious phylogenetic divergence from both historically recognized species (*interrogans* and *biflexa*). However, a new branch in the phylogenetic tree comprised by the sequence of *L. inadai* was also found (Hookey, 1993). Since this last strain bisected the pathogenic and saprophytic known clades, it was denominated “intermediate” (Hookey, 1993). Since then, these three genetic clades have been consistently obtained in the genomic classification of *Leptospira* spp. by using not only the *16S* DNA but other genomic markers including the whole genome (Fouts et al., 2016). Nonetheless, it is important to clarify that despite the phylogenetic division in three clades, the species belonging to both pathogenic and intermediate clades are considered “Infectious *Leptospira* spp.,” being both pathogenic in different mammal species including humans (Fouts et al., 2016). Hence, a more suitable classification for this clade has been suggested as pathogenic *Leptospira* spp. group II (Vincent et al., 2019). Indeed, some of the species from the “intermediate” or pathogenic *Leptospira* spp. group II clade have been increasingly found in febrile cases in humans (Levett et al., 2006; Chiriboga et al., 2015; Tsuboi et al., 2017; Puche et al., 2018) and causing disease in different animal species (Zakeri et al., 2010; Romero-Vivas et al., 2013). Hence, these reports highlight the relevance of studying this group of *Leptospira* spp. (intermediate or pathogenic group II) whose role in the epidemiology dynamic and maintenance of the disease, as well as the link between different host species and humans remain unclear.

Since the clinical signs of the disease are not pathognomonic, laboratory confirmation of leptospirosis is mandatory for definitive diagnosis (Musso and La Scola, 2013). The diagnostic methods include either direct or indirect detection (Marquez et al., 2017). Within the indirect methods, the microscopic agglutination test (MAT) is considered the gold-standard for serological diagnosis, although the ELISA methods have shown promising levels of sensitivity and specificity (Penna et al., 2017). However, there are several bottlenecks that complicate the use of serological methods including: time-consuming, the need to maintain live strains which can be easily mixed up as well as the fact that cultures can easily contaminate by saprophytic *Leptospira* spp. or other bacteria (Goris and Hartskeerl, 2014). In the case of the direct detection methods, the *Leptospira* spp. culture is not suitable for clinical diagnosis since *Leptospira* spp. are difficult to isolate because of its slow growth (∼13 weeks) with a low probability of recovery and a high rate of contamination (Nisansala et al., 2018). Immunofluorescence assays (IFAs) targeting the exposed surface of proteins, such as OmpL54, on intact leptospires have been also developed as diagnostic methods (Pinne and Haake, 2011). However, this method required the specific recognition of surface-exposed protein epitopes by OmpL54-specific antibodies mainly raised against recombinant proteins; hence, epitopes of native surface-exposed proteins may not be recognized (Pinne and Haake, 2011), making the IFA prompt to yield false-negative results (Goris and Hartskeerl, 2014).

In the molecular *era*, the advantages of real-time polymerase chain reaction (qPCR) technology have motivated its use for the diagnosis of leptospirosis on different clinical specimens including whole blood (Villumsen et al., 2012), serum (Smythe et al., 2002; Ooteman et al., 2004; Musso et al., 2014), urine (Miotto et al., 2018), kidney, liver, placenta (Fornazari et al., 2012), and other tissue samples. However, most of the validated qPCR assays for diagnostic purposes have been designed to target highly polymorphic genes such as *lipL32* (Stoddard, 2013) and *lfb1* (Merien et al., 2005) which detect only the pathogenic group I of *Leptospira* genus, failing in the recognition of the species from “intermediate” clade (Tsuboi et al., 2017; Thibeaux et al., 2018). This last issue has been suggested as a potential aspect that contributes to the under-report of *Leptospira* spp. in several geographic regions (Tsuboi et al., 2017). To overcome this problem, the current study was aimed to validate a multiplex qPCR to selectively detect infectious *Leptospira* spp. and differentially discriminate between pathogenic *Leptospira* spp. group I (pathogenic clade) and pathogenic *Leptospira* spp. group II (intermediate clade). The assay presented here was validated to target three specific regions of the *16S RNA* gene of the genome of *Leptospira* spp.: a particular region for the specific detection and discrimination of infectious *Leptospira* spp. and other two regions for the specific detection and discrimination of both pathogenic and intermediate groups (hereafter called pathogenic I and II, respectively). This novel multiplex assay enables a broader detection of the infectious *Leptospira* spp., representing an additional advantage for its use in epidemiological studies. The results obtained from the evaluation of the analytical and diagnostic parameters make the current validated assay suitable for diagnostic purposes.

## Materials and Methods

### Ethics Statement

No ethical approval was required as sampling was not undertaken by the authors.

### *Leptospira* spp. Strains and Samples

#### Strains

A total of 12 *Leptospira* spp. strains were used in the current study (Table 1). This collection included eight strains from the *Leptospira* spp. pathogenic group I, three strains from the *Leptospira* spp. pathogenic group II, and one strain from the saprophytic group (Table 1). In the cases of the strains *Leptospira inadai* and *Leptospira perolatii*, plasmids containing the genetic background of target regions for these strains were synthetized by IDT Integrated DNA technologies.

**TABLE 1:** Summary of microorganism used in this study.

**No.**	**Specie**	**Serovar (strain)**	**Source**	**qPCR multiplex (infectious/path-GI/path-GII)**
1	*Leptospira interrogans*	Autumnalis (Akiyami)	NVSL ATL-010	**Positive**/**positive**/negative
2	*Leptospira interrogans*	Bratislava (Jez Bratislava	NVSL ARL-050	**Positive**/**positive**/negative
3	*Leptospira interrogans*	Hardjo (Hardjoprajtino)	NVSL SJS-060	**Positive**/**positive**/negative
4	*Leptospira interrogans*	Canicola (Hond Utrecht IV)	NVSL CAL-010	**Positive**/**positive**/negative
5	*Leptospira interrogans*	Pomona (Pomona)	NVSL POS-010	**Positive**/**positive**/negative
6	*Leptospira interrogans (Icterohemorrhagiae)*	Copenhageni (M-20)	NVSL ICL-020	**Positive**/**positive**/negative
7	*Leptospira kirschneri*	Grippotyphosa (Andaman)	NVSL GRL-020	**Positive**/**positive**/negative
8	*Leptospira weilii*	celledoni	ATCC^®^ 43285^TM^	**Positive**/**positive**/negative
9	*Leptospira broomii*	–	ATCC^®^ BAA-1107^TM^	**Positive**/negative/**positive**
10	*Leptospira perolatii*^ϕ^	–	IDT Integrated DNA technologies	**Positive**/negative/**positive**
11	*Leptospira inadai*^ϕ^	–	IDT Integrated DNA technologies	**Positive**/negative/**positive**
12	*Leptospira biflexa*	patoc 1	ATCC^®^ 23582^TM^	Negative/negative/negative
13	*Salmonella enterica*	Typhimurium	ATCC^®^ 14028^TM^	Negative/negative/negative
14	*Escherichia coli*	Serotype O6, Biotype 1	ATCC^®^ 25922^TM^	Negative/negative/negative
15	*Escherichia coli*	–	ATCC^®^ 35218^TM^	Negative/negative/negative
16	*Listeria monocytogenes*	–	ATCC^®^ 7644^TM^	Negative/negative/negative
17	*Campylobacter jejuni*	–	ATCC^®^ 33291	Negative/negative/negative
18	*Chlamydia abortus^§^*	–	–	Negative/negative/negative
19	*Brucella abortus*	*–*	Heat killed culture kindly provided by Illinois Department of Agriculture Animal Disease Laboratory, Galesburg, IL	negative/negative/negative
20	*Brucella melitensis*		Heat killed culture kindly provided by Illinois Department of Agriculture Animal Disease Laboratory, Galesburg, IL	negative/negative/negative
21	*Brucella canis*		Culture stock from VDL at UIUC	negative/negative/negative
22	*Actinobacillus suis*	*–*	Culture stock provided by NVSL check test 2008	negative/negative/negative

*NVSL: National Veterinary Services Laboratories, United States Department of Agriculture (USDA), United States. ^ϕ^Plasmid containing the background sequence of *Leptospira perolatti* strain for the target regions was synthetized by IDT DNA technologies. ^§^ DNA kindly provided by Purdue University Animal Diseases Laboratory, West Lafayeette, IN, United States. VDL: Veterinary Diagnostic Laboratory. UIUC: University of Illinois at Urbana-Champaign. For a better Visualization, positive results were denoted in bold case.*

#### Samples

A total of 684 samples submitted for the molecular diagnosis of *Leptospira* spp. at Veterinary Diagnostic Laboratory (VDL) of the University of Illinois during the years 2012–2018 were used to evaluate the performance of the novel multiplex qPCR assay. The samples included urine, blood, kidney, placenta, liver, and fetal tissues (Supplementary Table S1) obtained from different animal species including wild and domestic animals (Supplementary Table S1).

### Assay Design

#### Sequence Dataset Selection and Analysis

A total of 59 sequences of *Leptospira* spp. available for the *rrs* gene (16S RNA) at GenBank Database, including 35 complete genome sequences were downloaded and used in the analysis (Supplementary Table S2). The dataset from the 35 complete genome sequences included: the 23 previously validly described *Leptospira* spp. and the 12 new *Leptospira* spp. recently proposed by Thibeaux et al. (2018).

Since Thibeaux et al. (2018) used a matrix-assisted laser desorption/ionization-time-of-flight (MALDI-TOF) mass spectrometry method (Rettinger et al., 2012) to classify the novel *Leptospira* spp. found (Thibeaux et al., 2018), a phylogenetic analysis using the complete gene of *16S RNA* was accomplished in the current study, to confirm the clade-based classification of these strains. The phylogenetic analysis was conducted following a Bayesian inference approach as described (Perez et al., 2012a). Based on the classification obtained from the phylogenetic analysis (Supplementary Figure S1), three groups of sequences were obtained and visually inspected to select the target regions by using multiple alignments with the algorithm Clustal W method included in the BioEdit program version 7.2.5 (Hall, 1999).

#### Primer and Probe Design

Different sets of primers and probes were designed with three main purposes (i) selective and differential targeting of infectious *Leptospira* spp. without detecting saprophytic *Leptospira* spp. Group, (ii) specific and differential detection of pathogenic group I, and (iii) specific and differential detection of pathogenic group II. All primers and probes were designed using the software Oligo 7.6 (Molecular Biology Insights, United States) as described (Acevedo et al., 2013). Briefly: the parameters were set to select the primer and probe for TaqMan assay by means of the priming efficiency of each primer and probe following the conditions optimized in Rios et al. (2018) with a subsequent *in silico* evaluation for the specificity using GenBank nr database and BLASTn searches as described by Rios et al. (2018). To avoid inefficient amplification reactions such as primer-dimer formation, all primers were also checked for their thermodynamic properties, secondary structures, and primer–primer interactions using the OligoExplorer v1.2 and the OligoAnalyzer v1.1.2 software programs (Gene Link^TM^, United States). Finally, the most promising primers/probe sets, to selectively detect infectious *Leptospira* spp. and discriminate between the different species of pathogenic group I and II of the *Leptospira* spp. were selected (Table 2).

**TABLE 2:** Primers and probe selected for the development of multiplex qPCR assay.

**Primer/probe**	**Sequence (5′-3′)**	**Set No. (*Leptospira* group targeted)**
infL-78F22	CGTGGGTAATCTTCCTCTGAGT	Set 1. (Infectious *Leptospira*)
InfL-264R2^a^	CTCAGTTCCATTGTGGCCGAA	
infL-179U20^b^	**^FAM–^**CTCGGAGAT/ **ZEN**/GAGCCCGCGTC**^–IABkFQ^**	
LP-1077F19	AGTTGGGCACTCGTAAGGA	Set 2. (*Leptospira* pathogenic group I)
LP-1203R23^a^	TTTTTGAGATTAGCTCCCCCTCG	
LP-1175U23^b^	**^Cy3–^**ACAATGGCC GGTACAAAGGGTAG**^–IAbRQ–SP^**	
LI-1044F20	GAGCGCAACCCCTATCGTAT	Set 3. (*Leptospira* pathogenic group II)
LI-1230R20^a^	CCAATCCGAACTGGGACCGA	
LI-1163U24^b^	^J^**^OE–^**CACACACGT/**ZEN**/ GCTACAATGGCCGAT**^–IABkFQ^**	

*^*a*^Reverse primer. ^*b*^Probe.*

#### Internal Control

In order to assess the presence of inhibitors or additional incidents that could take place during the DNA isolation procedure, a commercially available exogenous internal control (IC) was included in the assay. Thus, *Xeno*^TM^ *DNA control* (Thermo Fisher Scientific) was added to the samples during the DNA isolation step (section “Nucleic Acid Isolation”). This IC contains a unique artificial sequence that does not interfere with the sequence of the target and its amplification signal is detected on Cy5 channel.

### Nucleic Acid Isolation

#### DNA Isolation From Tissues, Blood, and Urine

Total nucleic acid was extracted by an automatic extraction protocol using KingFisher Flex (Thermofisher scientific) with MagMax CORE Nucleic Acid Purification Kit (Thermofisher scientific). In all cases, the manufacturer’s instructions were followed for each type of sample, briefly: for urine samples, 1.2 mL of urine was centrifuged at 6000 *g* for 10 min, the resulting pellet was re-suspended in 200 μL of 1X phosphate buffered saline (1X PBS), and used as starting material for the extraction process. In the case of tissue samples (kidney, placenta, liver), 25 mg of tissue was homogenized in 1 mL of 1X PBS using a Bead Bug Microtube Homogenizer (Benchmark Model D1030) at 3800 r/min during 1 min, 100 μL of this tissue homogenate was then used as starting material. In the case of blood samples, 200 μL was directly used in the extraction process.

All the starting materials were added to a 96-well S-block containing the bead mix (2:1 ratio by volume of magnetic beads and Proteinase K), and shaken on a platform shaker at 100–150 r/min for 2 min. Lysis and washing buffers were added according to the manufacturer’s instructions, 1 μL of the IC (approximately 2000 copies/μL) (see section “Internal Control”) was added during the extraction step. Finally, the DNA was extracted using MagMax Pathogen High Volume DW 96 protocol.

#### Standards Construction for Both Pathogenic Groups I and II of *Leptospira* spp.

Quantitative standards of *Leptospira* spp. genomic DNA were prepared from pure cultures of representative strains for each pathogenic group. Thus, DNA extracted from cultures of *L. interrogans* serovar Pomona and *Leptospira broomii* were quantified by a Qubit dsDNA broad range (2–1000 ng) Assay Kit in a Qubit 4.0 fluorometer (Invitrogen Corporation, Carlsbad, CA, United States), according to the manufacturer’s instructions; a sample volume of 1 μL was added to 199 μL of a Qubit working solution. Numbers of genomes were calculated assuming a *Leptospira* spp. genome size of 4.63 Mb by using the webtool http://scienceprimer.com/copy-number-calculator-for-realtime-pcr. Hence, a total of 1.3 × 10^7^ DNA copies/μL and 9.1 × 10^7^ DNA copies/μL were obtained for the standard from *L. interrogans* serovar Pomona and *L. broomii*, respectively.

### Simple qPCRs, Duplex-qPCR, and Multiplex System qPCR

All qPCR experiments were performed on an Applied Biosystems 7500 Fast Real-Time PCR System instrument (Thermo Fisher Scientific, Waltham, MA, United States). Through the optimization of the protocols, several experimental steps were accomplished to set up the reagent concentrations and the thermocycling parameters, briefly: three different concentrations of primers/probe [0.3 μM (forward and reverse primers)/0.15 μM (probe), 0.4 μM (forward and reverse primers)/0.20 μM (probe), and 0.5 μM (forward and reverse primers)/0.25 μM (probe)], and two thermocycling conditions (standard and fast two-step cycling) were assessed. Amplification efficiencies were calculated for each primers/probe set by standard curves using serial dilution from standards obtained, efficiency and standard errors were determined by employing Biogazelle qbase + software as described (Perez et al., 2017). A fast two-step cycling thermal profile (95°C for 30 s followed by 40 cycles of 95°C for 5 s, an annealing/elongation by 60°C for 20 s with acquisition of fluorescent data) was finally selected.

To assess the effect of interference of one set (primers/probe) over the other, the assays were first optimized in single format and finally optimized as a duplex-qPCR (for the detection of infectious *Leptospira* spp. and the detection of pathogenic group I)/simple-qPCR system (confirmation of pathogenic group II). Finally, all amplification reactions were conducted in final volume of 20 μL containing 5 μL of DNA template and the composition of the reaction was established as the lower concentration of primers and probes with the higher level of efficiency for the reaction (Table 3).

**TABLE 3:** Composition of mix for the qPCR multiplex assay for the detection of infectious *Leptospira* spp. and differentiation between both pathogenic groups I and II.

**qPCR multiplex**	**Reagent**	**Volume (μL)**	**Final concentration**	**Source**
Duplex	Primer infL-78F22	0.5	0.3 μM	IDT Integrated DNA technologies
	Primer InfL-264R2	0.5	0.3 μM	IDT Integrated DNA technologies
	Probe infL-179U20	0.25	0.15 μM	IDT Integrated DNA technologies
	Primer LP-1077F19	0.5	0.3 μM	IDT Integrated DNA technologies
	Primer LP-1203R23	0.5	0.3 μM	IDT Integrated DNA technologies
	Probe LP-1175U23	0.25	0.15 μM	IDT Integrated DNA technologies
	VetMAX^TM^ Xeno^TM^ IPC LIZ Assay	1	1X	Thermofisher Scientist
	PerfeCTa FastMix II Low ROX	10	1X	QuantaBio
	Nuclease free water	1.5	–	Thermofisher Scientist
	**∑**	**20**		
Single	LI-1044F20	0.5	0.3 μM	IDT Integrated DNA technologies
	LI-1230R20	0.5	0.3 μM	IDT Integrated DNA technologies
	LI-1163U24	0.25	0.15 μM	IDT Integrated DNA technologies
	VetMAX^TM^ Xeno^TM^ IPC LIZ Assay	1	1X	Thermofisher Scientist
	PerfeCTa FastMix II Low ROX	10	1X	QuantaBio
	Nuclease free water	2.75	–	Thermofisher Scientist
	**∑**	**20**		

### Limit of Quantification, Efficiency, and LIMIT of Detection

The limit of quantification (LoQ) was defined as the linear range of the assays and it was estimated from evaluating serial four-fold dilutions in nuclease-free water of quantified DNA standards obtained as described in 2.4.3; amplification efficiencies were determined as previously described by Perez et al. (Perez et al., 2017) employing Biogazelle qbase + software. The limit of detection (LoD) for the multiplex-qPCR assay was estimated by evaluating serial four-fold dilutions in nuclease-free water of quantified DNA standards obtained as described in the section “Standards Construction for Both Pathogenic Groups I and II of *Leptospira* spp.” The dilutions assessed ranged from 10^7^ RNA copies/μL to 10^–2^ DNA copies/μL for both *L. interrogans* serovar Pomona and *L. broomii*. Each dilution was tested in a set of 10 replicates. Thus, the LoD of the assays was determined using a Probit regression analysis implemented in the MedCalc Statistical Software version 19.0.7 (MedCalc Software bvba, Ostend, Belgium^1^ 2019). The LoD was defined as the concentration (copies of the gene/μL) that yielded 95% positive results along with the 95% confidence interval (CI) (Vaks et al., 2016).

### Analytical Sensitivity

Analytical sensitivity (ASe) was determined by dilution-to-extinction (DTE) experiments using four-fold serial dilutions of cultures of *L. interrogans*, serovar Pomona, and *L. broomii* into five different appropriate matrices. These matrices consisted of *Leptospira*-free tested bovine placenta tissue, canine urine, feline liver tissue, canine kidney tissue, and bovine blood. In addition, the matrix effect on the efficiency and sensitivity of the assays was also assessed. Like for the LoD, the Se for each clinical matrix was determined using a Probit regression analysis implemented in the MedCalc Statistical Software version 19.0.7 (MedCalc Software bvba, Ostend, Belgium^1^; 2019). The Se was defined as the concentration (copies of the gene/μL) that yielded 95% positive results along with a 95% CI (Vaks et al., 2016).

### Analytical Specificity

The specificity of the multiplex qPCR was assessed by the analysis of DNA of 12 *Leptospira* spp. included in the study and other non-*Leptospira* spp. causing disease with clinical signs resembling *Leptospira* spp. infection in different animal species (Table 1). All the samples were tested in triplicate by two different operators. In addition, all the amplification products obtained from the infectious *Leptospira* strains were purified from the agarose gel using QIAquick Gel Extraction Kit (Qiagen GmbH), and submitted for sequencing that was conducted under BigDyeTM terminator cycling conditions by an external laboratory ACGT Inc., Urbana, IL, United States. The quality of each sequence obtained was analyzed manually and the sequence similarity was checked against sequences deposited in the EMBL/GenBank using a BLAST search at NCBI site^2^. Sequences from each fragment amplified for each primer pair are available in Supplementary Text S2.

### Intra- and Inter-Assay Variability

The repeatability of the multiplex qPCR assay was calculated as reported by Rios et al. (2018), briefly: three different concentration of both *Leptospira* spp. strains *L. interrogans* serovar Pomona and *L. broomii* were used, strong positive (10^6^ DNA copies/μL), medium positive (10^4^ DNA copies/μL), and weak positive (10^2^ DNA copies/μL). The intra-assay variability was assessed by evaluating 20 replicates of each dilution, whereas the inter-assay variability was determined by analyzing each condition in independent runs conducted by two operators on different days. The coefficient of variation (CV) was obtained using the formula CV = SD [Ct-value]/overall mean[Ct-value] × 100, previously reported by Perez et al. (2012a).

### Interpretation of Results

Since non-specific amplifications from primer/primer or primer/probe interactions were not obtained after 55 cycles, a cut-off value for multiplex qPCR assay was established following the expression (1), to avoid miss amplifications or fluorescence artifacts. Thus, it was determined that all the samples yielding a cycle threshold (Ct) value below 40 (Ct < 40) were considered positive.

(1) $$C\,u\,t-o\,f\,f=C\,t_{L\,o\,D}+3\times S\,D_{\left(10\,x\,r\,e\,p\,l\,i\,c\,a\,t\,e\,s\,L\,o\,D\right)}$$

### Evaluation of the Diagnostic Performance

#### Comparison of the Performance on Clinical Samples

The performance of the new multiplex qPCR presented here on the 684 samples assessed (see section “Samples” and Supplementary Table S1) was compared with the results obtained from the qPCR assay routinely used for the diagnosis of *Leptospira* spp. at VDL (Smythe et al., 2002).

#### Bayesian Latent Class and Statistical Analysis

Bayesian methods have been increasingly used to assess the reliability of diagnostic tests in the absence of a true gold standard. In fact, Bayesian Latent class methods are currently suggested by the OIE for estimation of diagnostic sensitivity and specificity (OIE, 2014). Thus, the diagnostic performance of the new multiplex qPCR assay presented here was estimated by two different approaches without a perfect reference test (“without gold-standard”), (i) a two-test one-population Bayesian model defined by Li and Liu (2019) that allowed for a conditional covariance dependence (correlation) among tests for the evaluation of this particular model an in-house script was generated (Supplementary Text S1), and (ii) a three-test one-population Bayesian Latent class method based on Lahuerta-Marin et al. (2018) with two assays with conditional covariance dependence and the third one conditional independence, for this particular model the webtool Modelling of Infectious Disease Centre^3^ was used. For each model, a non-informative beta (1,1) prior was used. The data consisted of results obtained from the comparison of the new multiplex qPCR assay presented here and the qPCR assay routinely used at VDL for *Leptospira* spp. diagnosis (Smythe et al., 2002). Models were run in WinBUGS 1.4.3 (Lunn et al., 2000) with convergence estimates derived using 2,000,000 iterations of simulation with sampling done every 100 iterations. The first 5000 iterations were discarded as burn-in convergence was assessed by evaluation of the history, trace plots, and calculation of the Gelman-Rubin statistic diagnostic which compares within and between chain variability of the two chains that were run. Posterior medians with 95% probability intervals (PIs) corresponding to the 2.5th and 97.5th percentiles of the Monte Carlo sample were used to summarize parameter estimates of DSe and DSp for the assays compared.

The significant differences among the efficiency values from the different experimental conditions and matrices assessed were analyzed by a fit curve with non-linear regression, with 95% CIs of the parameters, using GraphPad Prism v8.3 (GraphPad software, Inc.). In all cases, a *p*-value < 0.05 was considered statistically significant.

#### Verification of the Performance on Clinical Samples

The accuracy of the predictions assessed for the different assays was also evaluated by sequencing the products obtained. The amplification products from the qPCR multiplex assays proposed here were cleaned and submitted for direct sequencing, whereas in the case of the amplicons obtained from the qPCR assay (Smythe et al., 2002) routinely used for the diagnosis of *Leptospira* spp. at VDL, the products were cleaned and cloned into the PGEM-t-easy vector (Promega, Madison WI, United States) following the manufacturer’s directions and read using the T7 and SP6 primers.

## Results

### The Establishment of the Multiplex qPCR Assay

#### *In silico* and *in vitro* Evaluation of the Target Regions Primers and Probe

**FIGURE 1:**
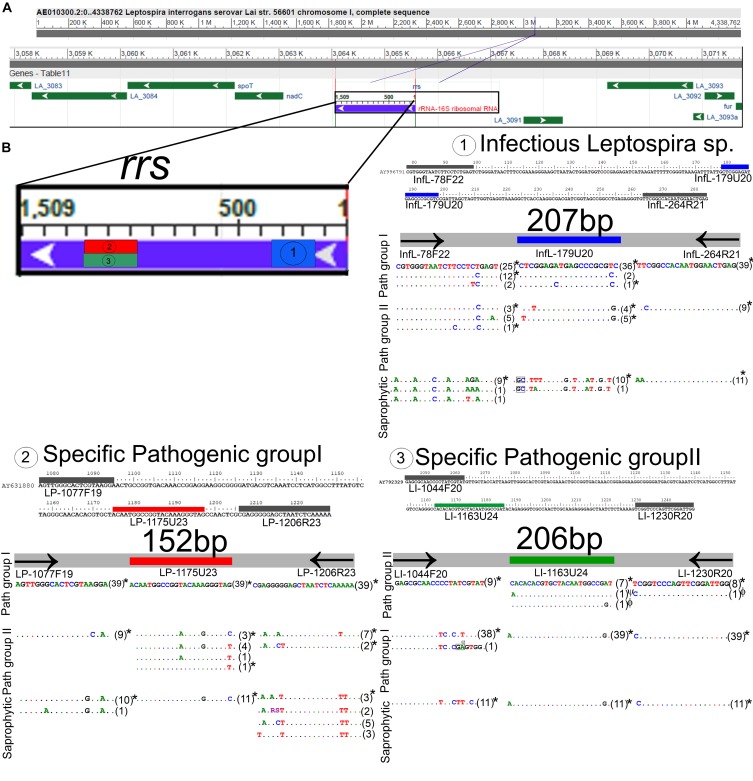
*In silico* analysis and locations of the target regions selected for the detection of *Leptospira* spp. **(A)** Whole genome representation of *Leptospira* spp., the targeted 16SRNA gene is denoted. **(B)** Location of the three target regions selected, the region targeted for the specific detection of infectious *Leptospira* spp. groups is denoted as 1 and highlighted in blue, the region targeted for the specific detection of pathogenic group I is denoted as 2 and highlighted in red, and the region targeted for the specific detection of pathogenic group I is denoted as 3 and highlighted in green; primer region analysis on all available sequences of *Leptospira* spp. (see Supplementary Table S1), for each target region the sequences of primers and probe are denoted, a total of 39 sequences for pathogenic group I, nine sequences for pathogenic group II and 11 sequences for saprophytic group were used. Dots indicate perfect match with the top sequence, letters indicate mismatches, insertions were denoted with black rectangles, all the polymorphisms tested using representative *Leptospira* spp. strains were denoted with an asterisk (^∗^), polymorphisms tested using genetic constructions were denoted with symbols (ψ,ϕ). The sizes for the amplicons of each target region are also denoted. The Whole-genome representation of *Leptospira* was used from the freely available NCBI source.

The development of new qPCR assays targeting the *16S RNA* gene for the diagnosis of *Leptospira* spp. has become a current need claimed by the scientific community (Tsuboi et al., 2017). This target region has been considered more suitable not only for clinical but also for epidemiological studies. Thus, to fit the “fitness of purpose” for the specific detection of infectious *Leptospira* spp. and further discrimination between both pathogenic groups, three target regions on the *16S RNA* gene were selected (Figures 1A,B).

For the specific detection of the whole infectious *Leptospira* spp. group, the primer pair/probe set selected (Table 2) was highly conserved for both pathogenic groups (<3 mismatches for all the sequences assessed) and highly discriminatory from the saprophytic group (Figure 1B). Thus, considering the 48 sequences belonging to infectious *Leptospira* spp. group, it was observed that on the region of the forward primer **infL-78F22**, 25 sequences from the *Leptospira* spp. pathogenic group I were 100% identical, 15 sequences (from both pathogenic group I and II) presented one mismatch (^18^C × T), two sequences from the pathogenic group I presented two mismatches (^17^T × C/^18^C × T), five sequence from the pathogenic group II presented two mismatches (^18^C × T/^21^A × G), and one sequence from the pathogenic group II also presented two mismatches (^18^C × T/^21^A × G) (Figure 1B). For the region targeted by the probe **infL-179U20**, it was found that 36 sequences from the pathogenic group I were 100% identical, two sequences from pathogenic group I presented one mismatch (^19^C × T), one sequence from the pathogenic group I presented two mismatches (^8^C × A/^19^C × T), four sequences from the pathogenic group II presented two mismatches (^3^T × G/^19^G × T), and five sequences from the pathogenic group II presented two other mismatches) (^1^T × C/^19^G × T) (Figure 1B). In the case of the region of the reverse primer **infL-264R21**, all the sequences from the pathogenic group I were 100% identical, and all the sequences from pathogenic group II presented one mismatch (^2^C × T) (Figure 1B).

For the specific detection of *Leptospira* spp. pathogenic group I, the primer pair/probe set selected (Table 2) was highly conserved for this specific group (Figure 1B). Indeed, the primers forward and reverse **LP-1077F19** and **LP1206R23** as well as the probe **LP-1175U23** showed a 100% sequence identity when compared to the 39 sequences assessed belonging to the *Leptospira* spp. pathogenic group I (Figure 1B). In addition, this primer pair and probe set was also highly discriminatory from *Leptospira* spp. pathogenic group II and the saprophytic group, showing more than five mismatches with all the sequences from both groups (Figure 1B).

Similarly, for the specific detection of *Leptospira* spp. pathogenic group II, the primer pair/probe set selected (Table 2) was highly conserved for this specific group (Figure 1B). The primer forward **LI-1044F20** showed a 100% sequence identity with all nine sequences assessed from *Leptospira* spp. pathogenic group II (Figure 1B). For the region targeted by the probe **LI-1163U24**, it was found that two sequences presented one mismatch (^1^A × C and ^23^G × A) (Figure 1B). In the case of the region of the reverse primer **LI-1230R20**, only one sequence from pathogenic group II presented one mismatch (^1^C × T) (Figure 1B). This primer pair/probe set was also highly discriminatory from *Leptospira* spp. pathogenic group I and the saprophytic group, showing more than five mismatches with all the sequences from both groups (Figure 1B).

**FIGURE 2:**
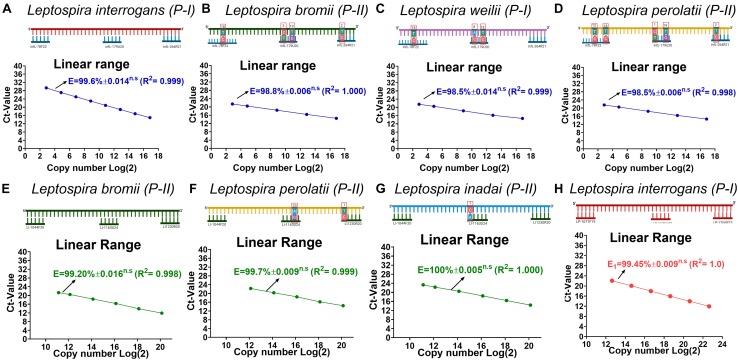
Evaluation of the effect on efficiency of the reaction caused by each mismatch found on each target region. **(A–D)** Evaluation of the effect on efficiency of the reaction for the target region of infectious *Leptospira* spp. group, **(E–G)** pathogenic group II, and **(H)** pathogenic group I. All polymorphisms denoted (^∗^) in Figure 1 were assessed and the efficiency values compared with 100% match for the primers/probe sets represented in **(A)** and **(D)** *L. interrogans* for infectious *Leptospira* group and pathogenic group I, **(B)** *L. bromii* for pathogenic group II. All the mismatches between the template target and the primers and probes are denoted highlighting the position and the replacement. All the *Leptospira* spp. strains used are denoted. The efficiency values, dynamic range, and linearity of the amplification reaction for the target regions are shown: **(A–D)** specific for the detection of infectious *Leptospira* spp. group denoted in blue, **(E–G)** specific for the detection of pathogenic group II denoted in green, and **(H)** *Leptospira* group I denoted in red. n.s: no statistically significant differences.

Since on the target regions for the primer pair/probe set for the specific detection of infectious *Leptospira* spp. and the discrimination of *Leptospira* spp. pathogenic group II some sequences presented a few mismatches (Figure 1B sequences denoted), we assessed the impact of these mismatches on the efficiency of detection of strains bearing these mismatches (Figure 2). The results obtained from the serial dilutions of DNA from these strains yielded no significant difference on the efficiency of the reaction among the different *Leptospira* spp. strains assessed (Figure 2). Thus, it was evidenced that the few mismatches found on the target region of the sequences of both pathogenic groups did not affect the detection and discrimination of infectious *Leptospira* spp. and *Leptospira* spp. pathogenic group II by the primers and probe selected.

#### Feasibility Study

**FIGURE 3:**
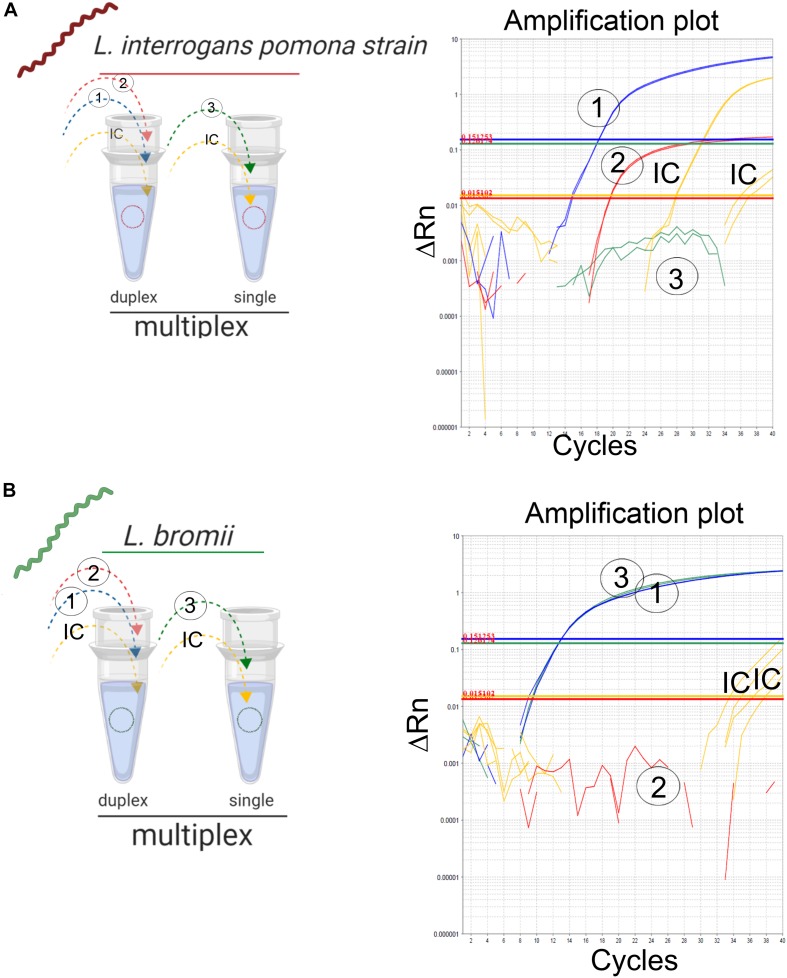
Detection of infectious *Leptospira* spp. group and differentiation of both pathogenic groups I and II. Schematic representation of the reactions and outcome of the qPCR multiplex assessed on different templates of *Leptospira* spp. **(A)** Pathogenic group I representative strain *L. interrogans* denoted in red and **(B)** pathogenic group II *L. bromii* denoted in green. Primers/probe sets for each target region were denoted, 1: infectious *Leptospira* spp. group (also denoted in blue), 2: pathogenic group I (also denoted in red), and 3: pathogenic group II (also denoted in green). Amplification reactions specific for each target region are denoted, 1: infectious *Leptospira* spp. group (also denoted in blue), 2: pathogenic group I (also denoted in red), and 3: pathogenic group II (also denoted in green). In all cases, IC: internal extraction control (also denoted in yellow).

The “fitness of purpose” of the assay presented here was the detection of infectious *Leptospira* spp. with the further discrimination between both pathogenic groups. A feasible approach is the implementation of a multiplex assay, which is intended to simplify the reaction, reduce the cost and time of execution (Rios et al., 2018). Since the primers/probe sets (Table 2, set 2 and 3) for the differentiation between both pathogenic groups spanned a common target region (Figure 1B), the combination of these primers and probes was not performed to avoid competition or interference. Thus, the two remaining possible combinations of primer/probe sets were assessed: the primers and probe for the specific amplification of infectious *Leptospira* spp. group, together with the primers for the specific detection of *Leptospira* spp. pathogenic group I (Table 2, set 1 and 2); and the primers and probe for the specific amplification of infectious *Leptospira* spp. group, together with the primers for the specific detection of *Leptospira* spp. pathogenic group II (Table 2, set 1 and 3). Both combinations yielded identical results, however, since the species from *Leptospira* spp. pathogenic group I have shown a higher prevalence than the species of *Leptospira* spp. pathogenic group II, for practical purposes, the combination (set1 + set2) was selected for the duplex (Figure 3A) and the set 3 was used in parallel for the conception of the multiplex assay (Figure 3B). Thus, signals of amplification from set 1, set 2, and the IC were obtained when the strain *L. interrogans*, serovar Pomona was used as reference material (Figure 3A) and subsequently, positive amplifications were obtained from the set 1, set 3, and the IC when the strain *L. bromii* was used (Figure 3B). Hence, each primer/probe set specifically recognized its corresponding target without interference, and no overlapped signals were obtained (Figure 3). The amplification of the IC did not affect the amplification of the specific targets (Figure 3). Hence, the multiplex assay was finally conceived as a duplex qPCR for the specific detection of infectious *Leptospira* spp. together with the discrimination of *Leptospira* spp. pathogenic group I (Figure 3A), with the amplification side-by-side on the same run of *Leptospira* spp. pathogenic group II (Figure 3B), and the IC in both reaction vessels (Figure 3).

### Optimization and Evaluation of Analytical Parameters

**FIGURE 4:**
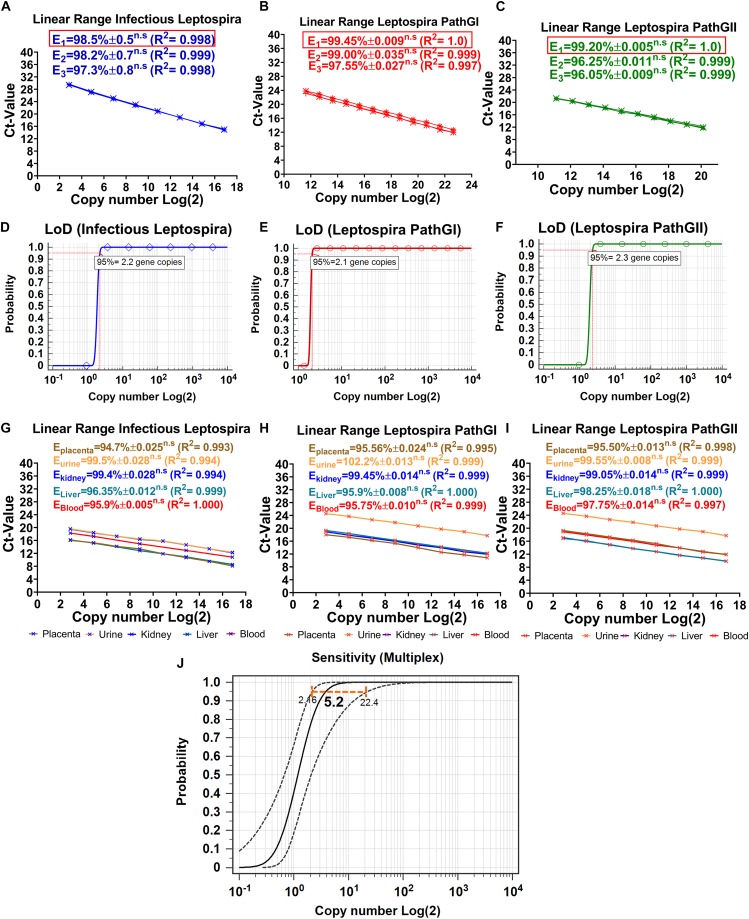
Dynamic range, limit of detection, matrix-effect, and analytical sensitivity of the novel multiplex qPCR assay. Evaluation of the different concentrations of primers and probes on efficiency and dynamic range of the reaction specific for the detection of **(A)** infectious *Leptospira* spp. group (also denoted in blue), **(B)** pathogenic group I (also denoted in red), and **(C)** pathogenic group II (also denoted in green). All the values of efficiency of the reaction are shown, the linearity of the reaction is also shown; in all cases, E_1_ was determined with 0.3 μM primers and 0.15 μM probe, E_2_ was determined with 0.4 μM primers and 0.2 μM probe, E_3_ was determined with 0.5 μM primers and 0.25 μM probe, n.s: no statistically significant differences. Evaluation of the LoD, probit function for each specific reaction is shown: **(D)** reaction targeting infectious *Leptospira* spp. group (also denoted in blue), **(E)** reaction targeting pathogenic group I (also denoted in red), and **(F)** reaction targeting pathogenic group II (also denoted in green); LoD value detected with 95% of confidence is shown. Evaluation of the matrix-effect on efficiency and the linearity of the reaction for each target region: **(G)** infectious *Leptospira* spp. group, **(H)** pathogenic group I, and **(I)** pathogenic group II. All the efficiency values for each matrix are shown, each matrix is denoted and highlighted (placenta: brown, urine: yellow, kidney: blue, liver: turquoise, blood: red). All the values of efficiency of the reaction are shown, the linearity of the reaction is also shown, n.s: no statistically significant differences. **(J)** Representative evaluation of analytical sensitivity (ASe) of the multiplex qPCR assay, 95% confidence interval estimated for kidney tissue homogenate matrix is shown (all the remaining matrices yielded same result), value of the ASe determined by probit function is highlighted in bold case.

Optimization is defined to evaluate and adjust the most critical parameters of the assay to ensure that its performance is best suited to the intended application (OIE, 2013). Since the efficiency of the reaction is a significant factor reflecting the capacity of the assay to duplicate the DNA target and it can be affected by non-specific interactions (primer-primer or primer-probe), this parameter was used as reference, to estimate the thermal profile and best concentrations of primers and probes. From the evaluation of three different concentrations of primers and probes, it was consistently obtained for the three targeted regions that the lower concentration of primers and probes showing the higher efficiency values was 0.3 μM for primers and 0.15 μM for probes (Figures 4A–C). When higher concentrations of primers and probes were assessed, no significant differences in the efficiencies of the reactions were obtained (Figures 4A–C). Thus, the composition of the reactions was finally established as described in Table 3.

#### Limit of Detection, Dynamic Range, and Limit of Quantification

The limit of detection of a PCR assay has been recently defined, as consensus, as the minimal concentration of nucleic acid that yields a positive result at least with 95% of confidence (Forootan et al., 2017). By evaluating 10 replicates for each DNA dilution and the application of a probit function, the LoD for each target region was determined. For the specific detection of infectious *Leptospira* spp. group, the LoD was 2.2 gene copies/reaction, for *Leptospira* spp. pathogenic group I, it was 2.1 gene copies/reaction, and for *Leptospira* spp. pathogenic group II, it was 2.3 gene copies/reaction (Figures 4D–F). Since for all the target regions the LoD was practically the same (∼3 gene copies/reaction) and no significant differences were obtained when the single qPCRs were compared with the duplex qPCR, this value was defined as the LoD for the multiplex assay.

The dynamic range has been defined in MIQE guidelines (Bustin et al., 2009) as the range over which a reaction is linear (Kralik and Ricchi, 2017), in other words, the range in which the target of interest can be accurately quantified. Kralik and Ricchi (2017) also suggested the LoQ in the molecular diagnosis of microorganisms to be set as the lowest quantifiable concentration of the target yielding a CV < 25%. Thus, the results obtained from the three target regions presented in the current assay showed a linear range spanning between_ENREF_25 6.5 × 10^6^ DNA copies/reaction to ∼9 DNA copies/reaction, with this value considered the LoQ for the multiplex assay (Figures 4G–I).

#### Matrix Effect and Analytical Sensitivity

The matrix samples usually include inhibitory factors that could interfere with the performance of PCR assays (Perez et al., 2012b); hence, the effect induced by the different matrices on which the assays are intended to be used must be assessed (OIE, 2013). As during the optimization for the multiplex qPCR assay, the effect of the different matrices on the performance of the multiplex qPCR assay was determined by estimating the deviation that the different matrices cause to the efficiency of the reactions (Figures 4G–I). No significant differences were obtained for any of the five matrices assessed for the three targeted regions (Figures 4G–I). In addition, all the efficiency values obtained were higher than 94% (E_matrix_ > 94%), making the qPCR multiplex suitable to be applied in these five matrices in diagnostic evaluations (Figures 4G–I).

Although the LoD is a measure of the ASe, these two parameters differ from each other, in the fact that the ASe is the minimal amount of the nucleic acid target that can be detected in a specific matrix at least with a 95% of confidence (OIE, 2013). Thus, the different components of the matrices could have an impact on the ASe, which could lead to obtaining different Ase for each biological matrix. From the evaluation of the ASe of the three target regions for each matrix assessed, no significant differences were found among them, the ASe for the three targeted regions was 5.2 copies/reaction 95% CI (2.16–22.4 gene copies/reaction) (Figure 4J). Thus, the qPCR multiplex assay was demonstrated to be highly sensitive.

#### Analytical Specificity

**FIGURE 5:**
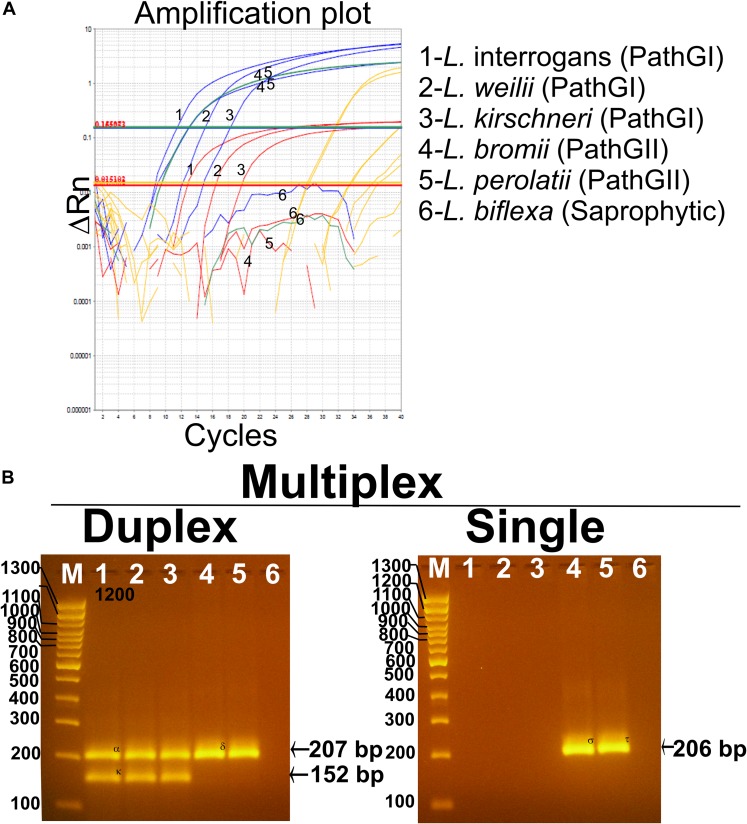
Performance of the multiplex qPCR assay on different *Leptospira* spp. strains. **(A)** Amplification curves for each sample using different *Leptospira* spp. strains included in pathogenic group I (*L. interrogans*, *L. weillii*, and *L. kirschineri*), pathogenic group II (*L. bromii* and *L. perolatii*), and saprophytic group (*L. biflexa*), also listed in Table 1. Amplification curves for the infectious *Leptospira* spp. group are shown in blue, amplification curves for the pathogenic group I are shown in red, amplification curves for the pathogenic group II are shown in green, amplification curves for internal control are shown in yellow, *Leptospira* spp. used as representative strains are denoted. **(B)** Confirmation of the amplification products resulting from the multiplex qPCR assay by electrophoresis on 3% agarose gel. In all case, 1—*L. interrogans*; 2—*L. weillii*; 3—*L. kirschineri*; 4—*L. bromii*; 5—*L. perolatti*; 6—*L. biflexa*; and M—molecular weight marker, 100 bp (Thermofisher scientific cat no. 15628019). Specific amplification product sizes are denoted: (infectious *Leptospira* spp. = 207 bp, pathogenic group I = 152 bp, and pathogenic group II = 206 bp). Confirmation by sequencing of the amplification products was also accomplished (see Supplementary Text S2).

The analytical specificity (ASp) of the Multiplex qPCR assay was determined considering the three major concepts defining this parameter: (i) inclusivity, (ii) exclusivity, and (iii) selectivity. The inclusivity of the assays was shown by the fact that the multiplex qPCR was able to specifically detect all the strains from infectious *Leptospira* spp. group (Table 1 and Figure 5), as well as to discriminate between the different strains from the pathogenic group I and the pathogenic group II (Table 1 and Figure 5). On the other hand, the exclusivity of the qPCR multiplex assays was evidenced since the assays did not amplify the nucleic acid from the *Leptospira* spp. saprophytic group (Table 1 and Figure 5) or any other non-*Leptospira* spp. related microorganism (Table 1). Likewise, the selectivity of the qPCR multiplex assay did not show amplification of any of the five biological matrices assessed (Supplementary Figure S2). Thus, the qPCR multiplex assay selectively detected the target without any interference by the matrices’ components.

#### Precision of the Multiplex qPCR Assay

Precision has been defined as the degree of agreement of measurements under specified conditions. In the practice, the precision of qPCR assays could be determined by two conditions termed repeatability and reproducibility (Kralik and Ricchi, 2017). For the case of the multiplex qPCR assay developed in the current study, we assessed the precision of the test determined by the repeatability intra and inter assay, considering within-laboratory variations. Thus, for the intra-assay repeatability, from 20 replicates included in the same experiment, the highest level of variability was obtained from the target region specific for the detection of infectious *Leptospira* spp. group, with a CV = 1.4% (Table 4). Hence, this value was considered as maximum intra-variability of the multiplex qPCR assay. In the case of the inter-assay repeatability, from the 20 replicates evaluated by two operators on different days, the highest level of variability was also obtained from the target region specific for the detection of infectious *Leptospira* spp. group, with a CV = 1.84% (Table 4). In all cases, a CV lower than 2.0% was obtained for all the targeted regions when intra and inter runs variabilities were assessed, this result is indicative of a highly repeatable and reproducible assay.

**TABLE 4:** Repeatability intra-assay and reproducibility inter-assay for the multiplex qPCR assay.

		**Mean Ct ± SD**			**CV**
		**10^7^ gene copies**	**10^4^ gene copies**	**10 gene copies**	
Multiplex qPCR Repeatability	Set 1	8.17 ± 0.04	15.49 ± 0.22	32.37 ± 0.09	0.27–1.40%
	Set 2	8.64 ± 0.04	15.98 ± 0.18	31.61 ± 0.43	1.15–1.37%
	Set 3	8.89 ± 0.02	16.37 ± 0.03	32.37 ± 0.09	0.20–1.31%
Multiplex qPCR Reproducibility	Set 1	8.11 ± 0.12	15.73 ± 0.29	32.47 ± 0.38	1.19–1.84%
	Set 2	8.48 ± 0.11	15.20 ± 0.21	31.67 ± 0.45	1.29–1.42%
	Set 3	8.87 ± 0.04	16.23 ± 0.08	32.09 ± 0.48	0.45–1.49%

*Ct: cycle threshold. SD: standard deviation. CV: coefficient of variation.*

### Diagnostic Performance of the qPCR Multiplex Assay

The results yielded by the multiplex qPCR assay in comparison with the qPCR assay for the diagnosis of *Leptospira* spp. (Smythe et al., 2002) (currently in use at VDL) on 684 clinical samples are summarized in Table 5. Whereas most of the outcome from both methods agree [621 out of 684 (90.7%)], detecting positive and negative samples to *Leptospira* spp., certain discrepancies were observed (Table 5). Thus, 41 (∼6%) samples resulted positive to *Leptospira* spp. by the new multiplex qPCR assay (34 samples tested positive for pathogenic group I and seven samples tested positive for pathogenic group II) and negative by the assay in use at VDL (Smythe et al., 2002), whereas 22 (3.2%) samples resulted positive to *Leptospira* spp. by the assay in use at VDL (Smythe et al., 2002) and yielded negative for the new multiplex qPCR assay.

**TABLE 5:** Performance of both qPCR on 684 clinical samples^1^ submitted at VDL suspected of *Leptospira* spp. infection.

**qPCR (Smythe et al., 2002) (Test A)**	**qPCR multiplex (Test B)**	**Count**
Negative	Negative	560
Negative	Positive	41
Positive	Negative	22
Positive	Positive	61
		**∑** = 684

*^1^Samples are listed in Supplementary Table S1.*

In order to get deep insight regarding the discrepancies showed by both diagnostic tests, Bayesian statistical analyses were performed. On the one hand, a clear limitation of classical latent class model comes from the identification of different populations with different prevalence (Lahuerta-Marin et al., 2018). However, more extended models have made suitable the use of one population with higher level of accuracy, those satisfying the condition of *Expression (2)* (Toft et al., 2005). Similarly, Li and Liu (2019) also proposed two simpler and feasible models to compare two tests on only one population in absence of a *gold standard* and under a conditional covariance dependency. Results obtained from the evaluation of the model proposed by Li and Liu (2019) available as WinBugs script (Supplementary Text S1), yielded values of diagnostic sensitivity of DSe_(TestA)_ = 96.45% and DSe_(TestB)_ = 96.66% and diagnostic specificity of DSp_(TestA)_ = 95.18% and DSp_(TestB)_ = 96.68%, where Test A is the qPCR assay in use at VDL (Smythe et al., 2002) and Test B the qPCR multiplex assays developed here (Supplementary Table S3). Thus, both diagnostic parameters for both assays were only slightly different, with a higher level of sensitivity and specificity for the new qPCR multiplex assay developed. However, after the confirmation of the amplification product by sequence analysis and considering these results obtained from sequencing as a third test, fulfilling the requirements of Expression (2), the values for both diagnostic parameters changed drastically (Table 6). A decrease in the value of DSe to 59.6% by the assay in use at VDL (Smythe et al., 2002) was observed, the DSp of this tests was not affected whereas both parameters for the qPCR multiplex assay developed here considerably improved with a DSe = 99.8% and a DSp = 100%. These results were mainly influenced by the fact that the 22 samples that tested positive by the qPCR assay reported by Smythe et al. (2002) and negative by the multiplex qPCR were identified by sequencing as *L. biflexa* and *Leptospira meyeri* from the saprophytic group (Supplementary Text S2). All 41 samples that were positive by the multiplex qPCR but not detected by the qPCR assay reported by Smythe et al. (2002) were identified as infectious *Leptospira* spp. In addition, this analysis determined a prevalence for infectious *Leptospira* spp. of 14.9%, from which the predictive values for the new assay revealed a high level of accuracy in the test (see Table 6, positive and negative predictive values). It is important to denote that, in all cases, all parameters were obtained from a high level of convergence of chain on the model used as shown in all the histograms obtained (Supplementary Figure S3).


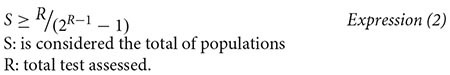


**TABLE 6:** Diagnostic parameters for the multiplex qPCR assay assessed by Bayesian Latent class analysis^§^.

**Parameters**	**Median (95%CI)**
Prevalence	14.9(12.3−17.8)
**Test A**	
DSe	59.6(49.6−68.8)
DSp	96.2(94.4−97.6)
PPV	73.2(63.0−82.1)
NPV	93.1(91.0−95.0)
**Test B**	
DSe	99.8(97.7−100)
DSp	100(99.6−100)
PPV	99.8(97.6−100)
NPV	100(99.6−100)
**Test C**	
DSe	99.8(97.6−100)
DSp	100(99.6−100)
PPV	99.8(97.5−100)
NPV	100(99.6−100)

*^§^The model described by Lim et al. (2013) and available online at http://mice.tropmedres.ac/publication.aspx was implemented. DSe: diagnostic sensitivity, DSp: diagnostic specificity, PPV: positive predictive value, NPV negative predictive value, Test A: qPCR (Smythe et al., 2002), Test B: qPCR multiplex presented here, Test C: sequencing analysis of the amplification products obtained.*

## Discussion

Leptospirosis represents a global threat for both human and animal health (Adler, 2015). In the recent years, the number of reports associating species from the pathogenic *Leptospira* spp. group II to cases of disease in humans (Levett et al., 2006; Chiriboga et al., 2015; Tsuboi et al., 2017; Puche et al., 2018) and animals (Zakeri et al., 2010; Romero-Vivas et al., 2013) has increased. This issue constitutes an additional concern to the complex epidemiology of this zoonotic agent, which is linked to more than 60,000 deaths in humans per year (Picardeau, 2017) and incalculable economic burden due to the cost of medical treatments (Torgerson et al., 2015) and livestock losses (Ellis, 2015). One of the major problems when establishing a proper control measure against *Leptospira* spp. is that the disease is basically underreported for many reasons, including difficulty in distinguishing clinical signs from those of other endemic diseases and the lack of appropriate diagnostic laboratory services. Despite that molecular methods, mainly those based on qPCR, have improved the diagnosis of *Leptospira* spp. in terms of cost, time, and reliability, most of the validated assays have been designed to target highly polymorphic genes such as *lipL32* (Stoddard, 2013), and *lfb1* (Merien et al., 2005) of the pathogenic group I, failing to detect species from the pathogenic group II (Tsuboi et al., 2017; Thibeaux et al., 2018). Hence, in the current study, we developed and validated a novel qPCR multiplex to enable the specific and selective detection of the whole group of infectious *Leptospira* spp. including both pathogenic groups I and II and moreover, selectively discriminate between them.

The use of *16S RNA* gene for the development of the current assay resulted in an advantageous target, enabling the detection of the whole group of infectious *Leptospira* spp. and the reliable differentiation between both groups (I and II), currently reclassified as P1 and P2 (Vincent et al., 2019). The fact that the *16S RNA* gene has been vastly supported as a good phylogenetic marker for *Leptospira* spp. classification (Fouts et al., 2016; Santos et al., 2018; Vincent et al., 2019) guarantees that this gene contains regions enabling the differentiation between the major groups of the *Letospira* spp. Moreover, using all sequences available at the GenBank database ensured that the design of the primers and probes in the current study had a broad and selective spectrum for the detection of all infectious *Leptospira* spp., with the exclusion of all saprophytic group and the novel clade of environmental *Leptospira* spp. with the species *idonii* as species type (Vincent et al., 2019). The format selected for the Taqman-type detection also holds additional advantages since its interpretation is straightforward, and also allows the combination of several wavelengths into the same vessel of reaction (Belak, 2007). The low impact showed by the mismatches found in the targeted regions on the efficiency of the amplification reactions is a clear indication that the multiplex assay validated has a high detection capacity. Thus, this assay will be highly useful not only for the diagnosis of the current known *Leptospira* spp. but also for additional strains that could emerge within both groups of interest (pathogenic I and II), since the assay is not affected by punctual changes in the sequences of the targets.

The incorporation of a qPCR method to the routine laboratory diagnostic can be fulfilled only if the new assay is rigorously validated through the proper standardization and optimization of all qPCR parameters (Bustin et al., 2009, 2013). Failing to comply with these critical aspects could trigger additional problems in the assay such as inadequate analytical parameters (sensitivity and specificity) (Perez and Diaz de Arce, 2009) and poor performance of the diagnostic parameters (Elnifro et al., 2000). Since the qPCR multiplex assay presented here was strictly optimized considering all the reaction parameters, including those related to system (Perez et al., 2011) and thermodynamic variables (Rios et al., 2018), the assay showed high levels of ASe and specificity, independently of the target region. Likewise, the efficiency of the reactions was consistently high regardless of the matrix sample assessed. It is relevant to clarify that the efficiency in the qPCR reactions is a critical parameter and it has been directly linked to three major aspects: (i) accurate design of the primers and probes with adequate size in the amplicon allowing a maximum yield that facilitates the detection of the target (Perez et al., 2017), (ii) appropriate optimization of the reaction parameters to avoid the amplification of undesirable targets (Acevedo et al., 2013), and (iii) the use of efficient system of nucleic acid isolation that limits the co-elution of macromolecules such as carbohydrates and proteins that could act as inhibitors of the amplification reaction (Suarez et al., 2007). In all cases, efficiency values higher than 94% were obtained, indicating that the three criteria were successfully accomplished, which also enables the use of the proposed assay on a broad spectrum of clinical samples.

It is also important to consider that the clinical presentation of leptospirosis is biphasic, the initial acute or septicemic phase lasts approximately about a week, followed by a subsequent immune phase, which is characterized by antibody production and excretion of leptospires in the urine (Levett, 2001). Although there is lack of studies uncovering the quantitative dynamic of *Leptsopira* spp. infections in animals, at least two studies have addressed this aspect in Leptospirosis infections in humans (Segura et al., 2005; Haake and Levett, 2015). From both studies, it can be inferred that during acute phase the bacterial load is around 10^7^ to 10^4^ Leptospires/mL (Segura et al., 2005; Haake and Levett, 2015), whereas in the immune phase leptospires were intermittently excreted to the lowest values of less than 10 Leptospires/mL (Segura et al., 2005). From this perspective and assuming that these values of bacterial load could be similar in the dynamic of infection of *Leptospira* spp. in animals, we can ensure that with the ASe shown by the new assay presented here, a broader window of detection for the agent at different stages of infection is guaranteed. On the other hand, leptospirosis is far from being a pathognomonic disease; therefore, a rigorous evaluation of the ASp is required mainly against those agents causing a similar clinical course. In the current study both suggested methods to evaluate the ASp (*in silico* and *in vitro*) (Broeders et al., 2014) were successfully accomplished revealing a congruence by both evaluations. Thus, the primers and probes selected, only recognized the specific targets for which the assay was intended. Indeed, the homologous and undesirable target, the *16S RNA* gene from saprophytic *Leptospira* spp. group, was not detected by the assay. Likewise, non-homologous but clinically related microorganisms were not detected by the new validated assay and the endogenous substances in clinical samples did not interfere with the outcome of the assay. The results obtained from the evaluation of the precision for the qPCR multiplex assay suggested that the level of repeatability and reproducibility of this new assay could facilitate its transference to different laboratories without a high impact in the results. However, to ensure which would be the deviation caused by using this new assay for different laboratories, a collaborative trial evaluation considering at least three different diagnostic laboratories should be accomplished in the future.

The evaluation of the performance of a qPCR assay on clinical field samples obtained from animals affected by the disease is a critical step for its use as accurate diagnostic tool (Rios et al., 2018). The evaluation of almost ∼700 samples from different hosts, collected during different years and different clinical specimens is one of the major strengths of the current validated assay. Because different species of *Leptospira* spp. tend to be more prevalent in one host than in others (Levett, 2001), using different samples from different hosts guaranteed that a wide spectrum of genetically different *Leptospira* spp. were assessed. In addition, the evaluation of different clinical specimens makes possible the detection of the agent at different stages of the disease.

The use of Bayesian latent class analysis represents a practical advantage, since it allows the evaluation of the performance of the assays without the use of a “perfect reference test” (OIE, 2014). This methodology also facilitates the estimation of DSe, DSp, and prevalence across all tests and populations without assuming the true status of the samples. It is also relevant to denote that although the model proposed by Li and Liu (2019) could be helpful in the practice and it is a less costly approach, this method could mask the values of DSe and DSp, as it was observed in the results obtained in the current study, which was evidenced by comparing the mentioned methodology with the three-test/one-populations approach. This last methodology yielded more accurate outcomes, at least in our evaluation; therefore, we encourage the application of this methodology during the validation process of new nucleic acid detection methods. In this regard, the three-test/one-populations method showed high levels of DSe and DSp for the new multiplex qPCR method validated here, evidencing a low DSe by the previous methods in use (Smythe et al., 2002). This result was mainly influenced by the outcome obtained from the sequence analysis, which revealed the assay reported by Smythe et al. (2002) recognized as positive the strains *L. biflexa* and *L. meyeri*. Since the authors claimed that the ASp for the primers/probe set was assessed against different non-pathogenic *Leptospira* strains (Smythe et al., 2002), this result was completely unexpected. However, the re-evaluation of the qPCR assay by Smythe et al. (2002) using the reference strain *L. biflexa* Patoc I from ATCC (Table 1) yielded a positive result confirming the results obtained from the clinical samples. Analyzing in detail the sequences of the primers/probe set reported by Smythe et al. (2002), it was observed that the forward primer presented five mismatches when aligned with the 11 sequences of the saprophytic group of *Leptospira*. However, the six nucleotides located toward the 3′-end showed a 100% match with the template sequence, thus this primer could perfectly hybridize in the DNA of the saprophytic group of *Leptospira* spp. with the mismatches acting as a T-tail allowing the amplification of these types of strains. Likewise, a single mismatch was observed in the probe sequence and this mismatch was not even located in a critical point. The same characteristic was observed on the reverse primer region (see Supplementary Figure S4). Thus, in agreement with the evidences found *in vitro*, the *in silico* evaluation revealed the primers/probe set reported by Smythe et al. (2002) likely amplifies species from the saprophytic group of *Leptospira* spp. as it was obtained during the evaluation of clinical samples. In the case of the new qPCR multiplex assay validated here, all the evaluations performed (*in silico*, *in vitro*, and from clinical samples) showed that the assay only targeted the infectious *Leptospira* spp. group with the additional differentiation of both pathogenic groups.

## Conclusion

The current study presents a novel validated multiplex qPCR assay for the specific detection of the infectious *Leptospira* spp. groups with the further differentiation between both pathogenic groups I and II. Since the new assay targets three specific regions within the *16RNA gene* of *Leptospira* spp., it enables a broader detection of the whole infectious group and segregates the saprophytic group. From the rigorous validation process, it was revealed that the assay proposed is sensitive, specific, and robust. The evaluation of the analytical and diagnostic parameters also showed that this new multiplex qPCR assay is a reliable diagnostic method for the detection of *Leptospira* spp.

## Author’s Note

LP is the guarantor of this work, had full access to all the data, and take full responsibility for the integrity of data and the accuracy of data analysis.

## Data Availability Statement

The raw data supporting the conclusions of this article will be made available by the authors, without undue reservation, to any qualified researcher.

## Author Contributions

LP, SL, and CM designed the research. LP performed the *in silico* analyses and wrote the manuscript. LP and SL performed the molecular biology analyses. VD performed the bacteriology analyses. LP, VD, SL, and CM analyzed and interpreted the data. SL, CM, and RF edited the manuscript and provided intellectual inputs. All the authors read and approved the final version of the manuscript.

## Conflict of Interest

The authors declare that the research was conducted in the absence of any commercial or financial relationships that could be construed as a potential conflict of interest.

## Footnotes

1 https://www.medcalc.org

2 http://www.ncbi.nlm.nih.gov

3 http://mice.tropmedres.ac/home.aspx

## Supplementary Material

FIGURE S1 Phylogenetic trees for *Leptospira* spp. Phylogenetic tree based on 16S RNA sequences using all non-redundant genomes available at GenBank. The main IBDV clades are denoted by designations and colors (Pathogenic: red, Intermediate: green and saprophytic: blue).

FIGURE S2 Performance of the multiplex qPCR assay on different matrices. In all cases, matrices of urine, liver, kidney, blood, and placenta were assessed. **(A)** Amplification curves for each sample using *L. interrogans* as positive control. **(B)** Amplification curves for each sample using *L. bromii* as positive control.

FIGURE S3 Evaluation of prevalence, sensitivities, specificities, and positive and negative predictive values (PPV and NPV) estimated by using an imperfect gold standard model (Bayesian latent class model). **(A, C, E, G, I, K**, and **M)** histogram for the distribution and **(B, D, F, H, J, L**, and **N)** trace plot for the MCMC chains. Evaluation for each parameter: prevalence, sensitivity, and specificity for each test is denoted. Test A: the qPCR assay in use at VDL (Smythe et al., 2002), Test B: Multiplex developed in the current study, and Test C: sequencing analysis of the amplification products obtained.

FIGURE S4 *In silico* analysis and locations of the target regions for the detection of *Leptospira* spp. for the primers and probe described by Smythe et al. (2002). Primers are denoted as arrow and probe as a green line, mismatches found in the sequences were denoted with red lines and the nucleotides are also shown.

TABLE S1 All clinical samples collected and assessed in the current study.

TABLE S2 Sequence of the 16S RNA of *Leptospira* spp. used in the study for the primer design.

TABLE S3 Sensitivity and specificity from Bayesian Latent class analysis.

## References

[B1] Acevedo A. M. Perera C. L. Vega A. Rios L. Coronado L. Relova D. (2013). A duplex SYBR Green I-based real-time RT-PCR assay for the simultaneous detection and differentiation of Massachusetts and non-Massachusetts serotypes of infectious bronchitis virus. *Mol. Cell. Probes* 27 184–192. 10.1016/j.mcp.2013.06.001 23810983

[B2] Adler B. (2015). History of leptospirosis and leptospira. *Curr. Top. Microbiol. Immunol.* 387 1–9. 10.1007/978-3-662-45059-8_1 25388129

[B3] Belak S. (2007). Molecular diagnosis of viral diseases, present trends and future aspects a view from the OIE collaborating centre for the application of polymerase chain reaction methods for diagnosis of viral diseases in veterinary medicine. *Vaccine* 25 5444–5452. 10.1016/j.vaccine.2006.11.068 17224207 PMC7115665

[B4] Broeders S. Huber I. Grohmann L. Berben G. Taverniers I. Mazzara M. (2014). Guidelines for validation of qualitative real-time PCR methods. *Trends Food Sci. Tech.* 37 115–126. 10.1016/j.tifs.2014.03.008

[B5] Bustin S. A. Benes V. Garson J. Hellemans J. Huggett J. Kubista M. (2013). The need for transparency and good practices in the qPCR literature. *Nat. Methods* 10 1063–1067. 10.1038/nmeth.2697 24173381

[B6] Bustin S. A. Benes V. Garson J. A. Hellemans J. Huggett J. Kubista M. (2009). The MIQE guidelines: minimum information for publication of quantitative real-time PCR experiments. *Clin. Chem.* 55 611–622. 10.1373/clinchem.2008.112797 19246619

[B7] Chiriboga J. Barragan V. Arroyo G. Sosa A. Birdsell D. N. Espana K. (2015). High prevalence of intermediate *Leptospira* spp. DNA in febrile humans from urban and rural ecuador. *Emerg. Infect. Dis.* 21 2141–2147. 10.3201/eid2112.140659 26583534 PMC4672404

[B8] Costa F. Hagan J. E. Calcagno J. Kane M. Torgerson P. Martinez-Silveira M. S. (2015). Global morbidity and mortality of Leptospirosis: a systematic review. *PLoS Negl. Trop. Dis.* 9:e0003898. 10.1371/journal.pntd.0003898 26379143 PMC4574773

[B9] Ellis W. A. (2015). Animal leptospirosis. *Curr. Top. Microbiol. Immunol.* 387 99–137. 10.1007/978-3-662-45059-8_6 25388134

[B10] Elnifro E. M. Ashshi A. M. Cooper R. J. Klapper P. E. (2000). Multiplex PCR: optimization and application in diagnostic virology. *Clin. Microbiol. Rev.* 13 559–570. 10.1128/cmr.13.4.559 11023957 PMC88949

[B11] Fairbrother J. M. (1984). Serological interrelationship of Leptospira serovar and genus-specific antigens by enzyme-linked immunosorbent assay. *J. Clin. Microbiol.* 20 1089–1093. 10.1128/jcm.20.6.1089-1093.1984 6084016 PMC271523

[B12] Fornazari F. da Silva R. C. Richini-Pereira V. B. Beserra H. E. Luvizotto M. C. Langoni H. (2012). Comparison of conventional PCR, quantitative PCR, bacteriological culture and the Warthin Starry technique to detect Leptospira spp. in kidney and liver samples from naturally infected sheep from Brazil. *J. Microbiol. Methods* 90 321–326. 10.1016/j.mimet.2012.06.005 22713608

[B13] Forootan A. Sjoback R. Bjorkman J. Sjogreen B. Linz L. Kubista M. (2017). Methods to determine limit of detection and limit of quantification in quantitative real-time PCR (qPCR). *Biomol. Detect. Quantif.* 12 1–6. 10.1016/j.bdq.2017.04.001 28702366 PMC5496743

[B14] Fouts D. E. Matthias M. A. Adhikarla H. Adler B. Amorim-Santos L. Berg D. E. (2016). what makes a bacterial species pathogenic?:comparative genomic analysis Of the genus *Leptospira*. *PLoS Negl. Trop. Dis.* 10:e0004403. 10.1371/journal.pntd.0004403 26890609 PMC4758666

[B15] Goris M. G. A. Hartskeerl R. A. (2014). Leptospirosis serodiagnosis by the microscopic agglutination test. *Curr. Prot. Microbiol.* 32 12E.5.1-12E.5.18. 10.1002/9780471729259.mc12e05s32 24510846

[B16] Haake D. A. Levett P. N. (2015). Leptospirosis in humans. *Curr. Top. Microbiol. Immunol.* 387 65–97. 10.1007/978-3-662-45059-8_5 25388133 PMC4442676

[B17] Hall T. A. (1999). BioEdit: a user-friendly biological sequence alignment editor and analysis program for windows 95/98/NT. *Nucleic Acids Symp. Series* 41 95–98.

[B18] Hookey J. V. (1993). Characterization of Leptospiraceae by 16S DNA restriction fragment length polymorphisms. *J. Gen. Microbiol.* 139 1681–1689. 10.1099/00221287-139-8-1681 7691982

[B19] Johnson R. C. Harris V. G. (1967). Differentiation of pathogenic and saprophytic letospires. I. growth at low temperatures. *J. Bacteriol.* 94 27–31. 10.1128/jb.94.1.27-31.1967 6027998 PMC251866

[B20] Johnson R. C. Livermore B. P. Walby J. K. Jenkin H. M. (1970). Lipids of parasitic and saprophytic leptospires. *Infect. Immun.* 2 286–291. 10.1128/iai.2.3.286-291.1970 16557833 PMC416003

[B21] Johnson R. C. Rogers P. (1964). Differentiation of pathogenic and saprophytic leptospires with 8-azaguanine. *J. Bacteriol.* 88 1618–1623. 10.1128/jb.88.6.1618-1623.1964 14244050 PMC277463

[B22] Kralik P. Ricchi M. (2017). A basic guide to real time PCR in microbial diagnostics: definitions. parameters, and everything. *Front. Microbiol.* 8:108. 10.3389/fmicb.2017.00108 28210243 PMC5288344

[B23] Lahuerta-Marin A. Milne M. G. McNair J. Skuce R. A. McBride S. H. Menzies F. D. (2018). Bayesian latent class estimation of sensitivity and specificity parameters of diagnostic tests for bovine tuberculosis in chronically infected herds in Northern Ireland. *Vet. J.* 238 15–21. 10.1016/j.tvjl.2018.04.019 30103911

[B24] Levett P. N. (2001). Leptospirosis. *Clin. Microbiol. Rev.* 14 296–326. 10.1128/CMR.14.2.296-326.2001 11292640 PMC88975

[B25] Levett P. N. Morey R. E. Galloway R. L. Steigerwalt A. G. (2006). Leptospira broomii sp. nov., isolated from humans with leptospirosis. *Int. J. Syst. Evol. Microbiol.* 56(Pt 3), 671–673. 10.1099/ijs.0.63783-63780 16514048

[B26] Levett P. N. Picardeau M. (2018). International committee on systematics of prokaryotes subcommittee on the taxonomy of *Leptospiraceae*. minutes of the closed meeting, 28 November 2017, palmerston North, New Zealand. *Int. J. Syst. Evol. Microbiol.* 68:3362. 10.1099/ijsem.0.002961 30095389 PMC6978982

[B27] Li T. Liu P. (2019). Comparison of two Bayesian methods in evaluation of the absence of the gold standard diagnostic tests. *Biomed. Res. Int.* 2019:1374748. 10.1155/2019/1374748 31531344 PMC6720053

[B28] Lim C. Wannapinij P. White L. Day N. P. Cooper B. S. Peacock S. J. (2013). Using a web-based application to define the accuracy of diagnostic tests when the gold standard is imperfect. *PLoS One* 8:e79489. 10.1371/journal.pone.0079489 24265775 PMC3827152

[B29] Lunn D. J. Thomas A. Best N. Spiegelhalter D. (2000). WinBUGS - A Bayesian modelling framework: concepts, structure, and extensibility. *Stat. Comp.* 10 325–337. 10.1023/a:1008929526011

[B30] Marquez A. Djelouadji Z. Lattard V. Kodjo A. (2017). Overview of laboratory methods to diagnose Leptospirosis and to identify and to type leptospires. *Int. Microbiol.* 20 184–193. 10.2436/20.1501.01.302 29529330

[B31] Merien F. Portnoi D. Bourhy P. Charavay F. Berlioz-Arthaud A. Baranton G. (2005). A rapid and quantitative method for the detection of *Leptospira* species in human leptospirosis. *FEMS Microbiol. Lett.* 249 139–147. 10.1016/j.femsle.2005.06.011 16006065

[B32] Miotto B. A. da Hora A. S. Taniwaki S. A. Brandao P. E. Heinemann M. B. Hagiwara M. K. (2018). Development and validation of a modified TaqMan based real-time PCR assay targeting the lipl32 gene for detection of pathogenic *Leptospira* in canine urine samples. *Braz. J. Microbiol.* 49 584–590. 10.1016/j.bjm.2017.09.004 29233483 PMC6066729

[B33] Musso D. La Scola B. (2013). Laboratory diagnosis of leptospirosis: a challenge. *J. Microbiol. Immunol. Infect.* 46 245–252. 10.1016/j.jmii.2013.03.001 23639380

[B34] Musso D. Roche C. Marfel M. Bel M. Nilles E. J. Cao-Lormeau V. M. (2014). Improvement of leptospirosis surveillance in remote Pacific islands using serum spotted on filter paper. *Int. J. Infect. Dis.* 20 74–76. 10.1016/j.ijid.2013.12.002 24384412

[B35] Nisansala G. G. T. Muthusinghe D. Gunasekara T. Weerasekera M. M. Fernando S. S. N. Ranasinghe K. N. P. (2018). Isolation and characterization of *Leptospira* interrogans from two patients with leptospirosis in Western Province. *Sri Lanka. J. Med. Microbiol.* 67 1249–1252. 10.1099/jmm.0.000800 30024367

[B36] OIE, (2013). *Principles and Methods of Validation of Diagnostic Assays for Infectious Diseases in: OIE Manual of Diagnostic Tests and Vaccines.* Paris: Office International des Epizooties.

[B37] OIE, (2014). *Statistical Approaches to Validation: in Manual of Diagnostic Tests and Vaccines for Terrestrial Animals. (2019).* Paris: OIE.

[B38] Ooteman M. C. Vago A. R. Koury M. C. (2004). Potential application of low-stringency single specific primer-PCR in the identification of Leptospira in the serum of patients with suspected leptospirosis. *Can. J. Microbiol.* 50 1073–1079. 10.1139/w04-096 15714239

[B39] Paiva-Cardoso M. Arent Z. Gilmore C. Hartskeerl R. Ellis W. A. (2013). Altodouro, a new *Leptospira serovar* of the Pomona serogroup isolated from rodents in northern Portugal. *Infect. Genet. Evol.* 13 211–217. 10.1016/j.meegid.2012.09.013 23070280

[B40] Penna B. Marassi C. D. Libonati H. Narduche L. Lilenbaum W. Bourhy P. (2017). Diagnostic accuracy of an in-house ELISA using the intermediate species *Leptospira fainei* as antigen for diagnosis of acute leptospirosis in dogs. *Comp. Immunol. Microbiol. Infect. Dis.* 50 13–15. 10.1016/j.cimid.2016.11.004 28131372

[B41] Perez L. J. Diaz de Arce H. (2009). A RT-PCR assay for the detection of encephalomycarditis virus infections in pigs. *Braz. J. Microbiol.* 40 988–993. 10.1590/S1517-838220090004000034 24031451 PMC3768574

[B42] Perez L. J. Diaz de Arce H. Cilloni F. Salviato A. Marciano S. Perera C. L. (2012a). An SYBR Green-based real-time RT-PCR assay for the detection of H5 hemagglutinin subtype avian influenza virus. *Mol. Cell. Probes* 26 137–145. 10.1016/j.mcp.2012.02.001 22421464

[B43] Perez L. J. Perera C. L. Frias M. T. Nunez J. I. Ganges L. de Arce H. D. (2012b). A multiple SYBR Green I-based real-time PCR system for the simultaneous detection of porcine circovirus type 2, porcine parvovirus, pseudorabies virus and Torque teno sus virus 1 and 2 in pigs. *J. Virol. Methods* 179 233–241. 10.1016/j.jviromet.2011.11.009 22119629

[B44] Perez L. J. Diaz de Arce H. Tarradas J. Rosell R. Perera C. L. Munoz M. (2011). Development and validation of a novel SYBR Green real-time RT-PCR assay for the detection of classical swine fever virus evaluated on different real-time PCR platforms. *J. Virol. Methods* 174 53–59. 10.1016/j.jviromet.2011.03.022 21458490

[B45] Perez L. J. Rios L. Trivedi P. D’Souza K. Cowie A. Nzirorera C. (2017). Validation of optimal reference genes for quantitative real time PCR in muscle and adipose tissue for obesity and diabetes research. *Sci. Rep.* 7:3612. 10.1038/s41598-017-03730-3739 28620170 PMC5472619

[B46] Picardeau M. (2017). Virulence of the zoonotic agent of leptospirosis: still terra incognita? *Nat. Rev. Microbiol.* 15 297–307. 10.1038/nrmicro.2017.5 28260786

[B47] Pinne M. Haake D. (2011). Immuno-fluorescence assay of leptospiral surface-exposed proteins. *J. Vis. Exp.* 53:e2805. 10.3791/2805 21750491 PMC3196178

[B48] Puche R. Ferres I. Caraballo L. Rangel Y. Picardeau M. Takiff H. (2018). *Leptospira venezuelensis* sp. nov., a new member of the intermediate group isolated from rodents, cattle and humans. *Int. J. Syst. Evol. Microbiol.* 68 513–517. 10.1099/ijsem.0.002528 29239713

[B49] Ramadass P. Jarvis B. D. Corner R. J. Cinco M. Marshall R. B. (1990a). DNA relatedness among strains of *Leptospira biflexa*. *Int. J. Syst. Bacteriol.* 40 231–235. 10.1099/00207713-40-3-231 2397191

[B50] Ramadass P. Marshall R. B. Jarvis B. D. (1990b). Species differentiation of Leptospira interrogans serovar hardjo strain Hardjobovis from strain Hardjoprajitno by DNA slot blot hybridisation. *Res. Vet. Sci.* 49 194–197. 10.1016/s0034-5288(18)31076-2 2236916

[B51] Ramadass P. Jarvis B. D. Corner R. J. Penny D. Marshall R. B. (1992). Genetic characterization of pathogenic *Leptospira* species by DNA hybridization. *Int. J. Syst. Bacteriol.* 42 215–219. 10.1099/00207713-42-2-215 1581182

[B52] Rettinger A. Krupka I. Grunwald K. Dyachenko V. Fingerle V. Konrad R. (2012). Leptospira spp. strain identification by MALDI TOF MS is an equivalent tool to 16S rRNA gene sequencing and multi locus sequence typing (MLST). *BMC Microbiol.* 12:185. 10.1186/1471-2180-12-185 22925589 PMC3460781

[B53] Rios L. Perera C. L. Coronado L. Relova D. Alvarez A. M. Ganges L. (2018). Multi-target strategy for pan/foot-and-mouth disease virus (FMDV) detection: a combination of sequences analysis, *in silico* predictions and laboratory diagnostic evaluation. *Front. Vet. Sci.* 5:160. 10.3389/fvets.2018.00160 30050913 PMC6052897

[B54] Romero-Vivas C. M. Cuello-Perez M. Agudelo-Florez P. Thiry D. Levett P. N. Falconar A. K. (2013). Cross-sectional study of Leptospira seroprevalence in humans, rats, mice, and dogs in a main tropical sea-port city. *Am. J. Trop. Med. Hyg.* 88 178–183. 10.4269/ajtmh.2012.12-0232 23149584 PMC3541732

[B55] Santos L. A. Adhikarla H. Yan X. Wang Z. Fouts D. E. Vinetz J. M. (2018). Genomic comparison among global isolates of *L. interrogans* serovars copenhageni and icterohaemorrhagiae identified natural genetic variation caused by an indel. *Front. Cell Infect. Microbiol.* 8:193. 10.3389/fcimb.2018.00193 29971217 PMC6018220

[B56] Segura E. R. Ganoza C. A. Campos K. Ricaldi J. N. Torres S. Silva H. (2005). Clinical spectrum of pulmonary involvement in leptospirosis in a region of endemicity, with quantification of leptospiral burden. *Clin. Infect. Dis.* 40 343–351. 10.1086/427110 15668855 PMC2366057

[B57] Smythe L. D. Smith I. L. Smith G. A. Dohnt M. F. Symonds M. L. Barnett L. J. (2002). A quantitative PCR (TaqMan) assay for pathogenic *Leptospira* spp. *BMC Infect. Dis.* 2:13. 10.1186/1471-2334-2-13 12100734 PMC117785

[B58] Stobart Gallagher M. A. Dunn N. (2019). *Leptospirosis (Weil Disease).* Treasure Island: StatPearls.

[B59] Stoddard R. A. (2013). Detection of pathogenic *Leptospira* spp. through real-time PCR (qPCR) targeting the LipL32 gene. *Methods Mol. Biol.* 943 257–266. 10.1007/978-1-60327-353-4_17 23104295

[B60] Suarez D. L. Das A. Ellis E. (2007). Review of rapid molecular diagnostic tools for avian influenza virus. *Avian. Dis.* 51(Suppl.), 201–208. 10.1637/7732-101006-REGR.1 17494554

[B61] Thibeaux R. Girault D. Bierque E. Soupe-Gilbert M. E. Rettinger A. Douyere A. (2018). Biodiversity of environmental leptospira: improving identification and revisiting the diagnosis. *Front. Microbiol.* 9:816. 10.3389/fmicb.2018.00816 29765361 PMC5938396

[B62] Toft N. Jorgensen E. Hojsgaard S. (2005). Diagnosing diagnostic tests: evaluating the assumptions underlying the estimation of sensitivity and specificity in the absence of a gold standard. *Prev. Vet. Med.* 68 19–33. 10.1016/j.prevetmed.2005.01.006 15795013

[B63] Torgerson P. R. Hagan J. E. Costa F. Calcagno J. Kane M. Martinez-Silveira M. S. (2015). Global burden of leptospirosis: estimated in terms of disability adjusted life years. *PLoS Negl. Trop. Dis.* 9:e0004122. 10.1371/journal.pntd.0004122 26431366 PMC4591975

[B64] Tsuboi M. Koizumi N. Hayakawa K. Kanagawa S. Ohmagari N. Kato Y. (2017). Imported *Leptospira* licerasiae infection in traveler RETURNING to Japan from Brazil. *Emerg. Infect. Dis.* 23 548–549. 10.3201/eid2303.161262 28221126 PMC5382744

[B65] Vaks J. E. Hemyari P. Rullkoetter M. Santulli M. J. Schoenbrunner N. (2016). Verification of claimed limit of detection in molecular diagnostics. *J. Appl. Lab. Med.* 1 260–270. 10.1373/jalm.2016.020735 33626838

[B66] Valverde Mde L. Goris M. G. Gonzalez V. Anchia M. E. Diaz P. Ahmed A. (2013). New serovars of *Leptospira* isolated from patients in Costa Rica: implications for public health. *J. Med. Microbiol.* 62(Pt 9), 1263–1271. 10.1099/jmm.0.058545-58540 23788592

[B67] Villumsen S. Pedersen R. Borre M. B. Ahrens P. Jensen J. S. Krogfelt K. A. (2012). Novel TaqMan(R) PCR for detection of *Leptospira* species in urine and blood: pit-falls of in silico validation. *J. Microbiol. Methods* 91 184–190. 10.1016/j.mimet.2012.06.009 22750039

[B68] Vincent A. T. Schiettekatte O. Goarant C. Neela V. K. Bernet E. Thibeaux R. (2019). Revisiting the taxonomy and evolution of pathogenicity of the genus *Leptospira* through the prism of genomics. *PLoS Negl. Trop. Dis.* 13:e0007270. 10.1371/journal.pntd.0007270 31120895 PMC6532842

[B69] Vk C. Ty L. Wf L. Ywy W. S. An S. S Z. (2018). Leptospirosis in human: biomarkers in host immune responses. *Microbiol. Res.* 207 108–115. 10.1016/j.micres.2017.11.015 29458845

[B70] Zakeri S. Khorami N. Ganji Z. F. Sepahian N. Malmasi A. A. Gouya M. M. (2010). Leptospira wolffii, a potential new pathogenic Leptospira species detected in human, sheep and dog. *Infect. Genet. Evol.* 10 273–277. 10.1016/j.meegid.2010.01.001 20074666

