# Supplementary figures and images for: A Validated Multiplex Real-Time PCR Assay for the Diagnosis of Infectious Leptospira spp.: A Novel Assay for the Detection and Differentiation of Strains From Both Pathogenic Groups I and II
Source: Front Microbiol. 2020 Mar 20;11:457. doi: 10.3389/fmicb.2020.00457 (PMC7100377; doi:10.3389/fmicb.2020.00457)

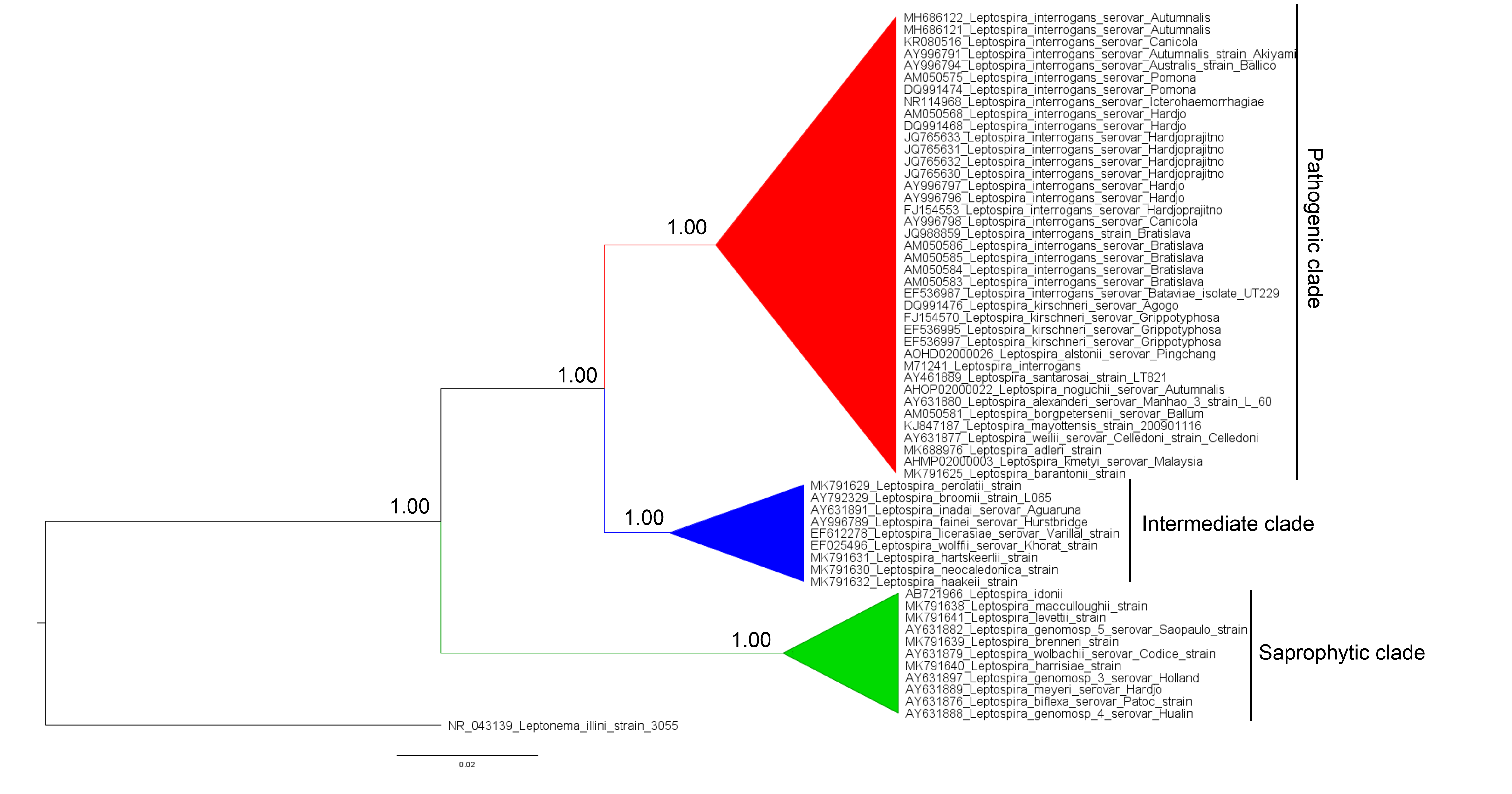

Supplement: FIGURE S1 — Phylogenetic trees for Leptospira spp. Phylogenetic tree based on 16S RNA sequences using all non-redundant genomes available at GenBank. The main IBDV clades are denoted by designations and colors (Pathogenic: red, Intermediate: green and saprophytic: blue). [file Image_1.TIF]

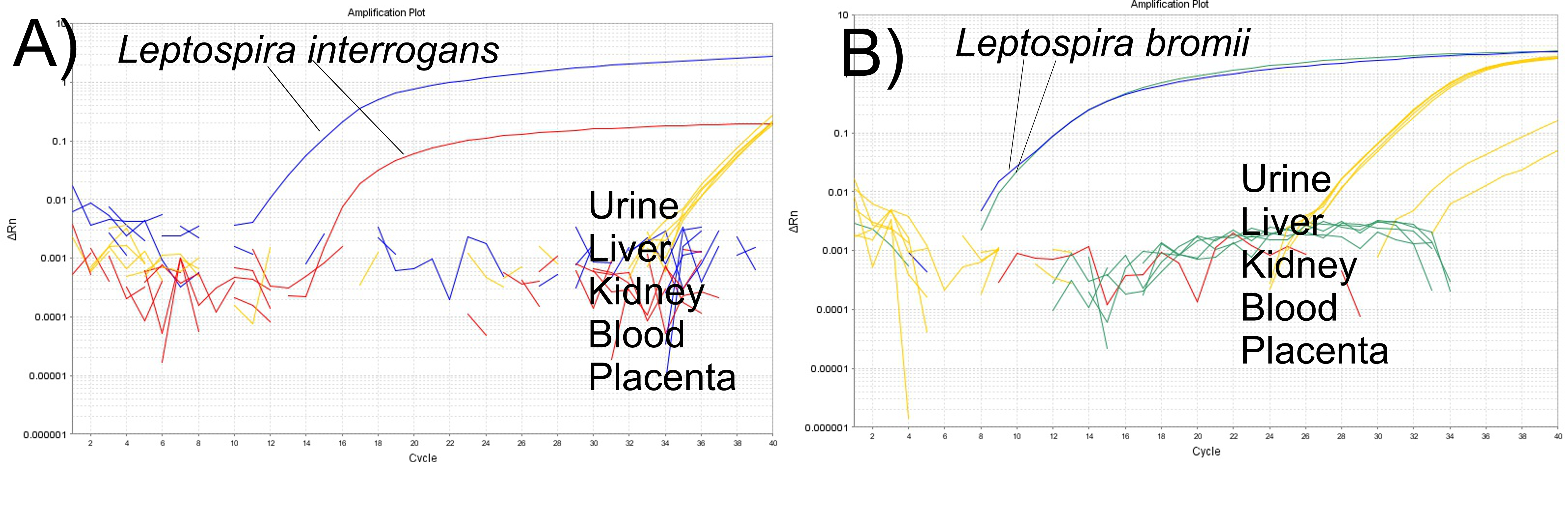

Supplement: FIGURE S2 — Performance of the multiplex qPCR assay on different matrices. In all cases, matrices of urine, liver, kidney, blood, and placenta were assessed. (A) Amplification curves for each sample using L. interrogans as positive control. (B) Amplification curves for each sample using L. bromii as positive control. [file Image_2.TIF]

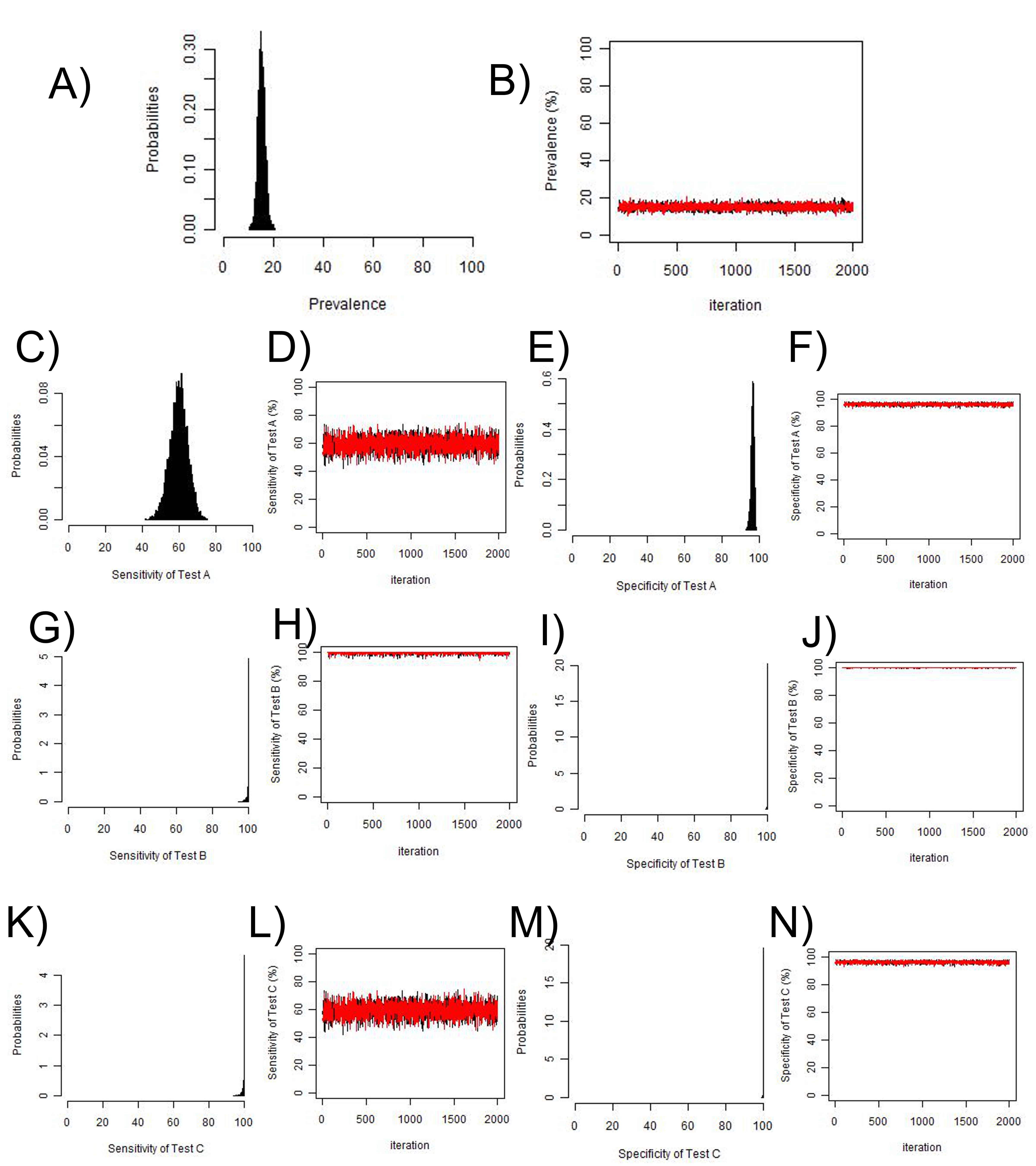

Supplement: FIGURE S3 — Evaluation of prevalence, sensitivities, specificities, and positive and negative predictive values (PPV and NPV) estimated by using an imperfect gold standard model (Bayesian latent class model). (A, C, E, G, I, K, and M) histogram for the distribution and (B, D, F, H, J, L, and N) trace plot for the MCMC chains. Evaluation for each parameter: prevalence, sensitivity, and specificity for each test is denoted. Test A: the qPCR assay in use at VDL (Smythe et al., 2002), Test B: Multiplex developed in the current study, and Test C: sequencing analysis of the amplification products obtained. [file Image_3.TIF]

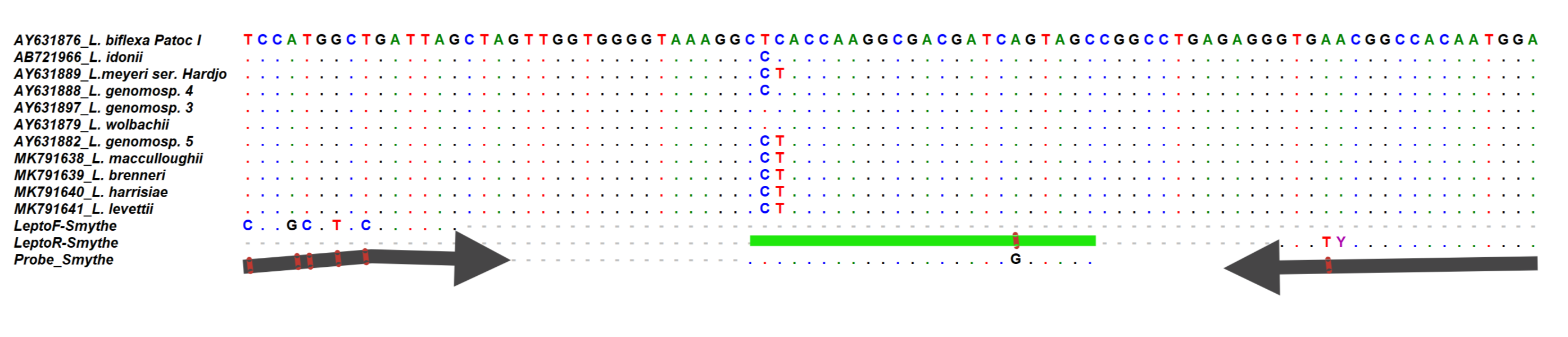

Supplement: FIGURE S4 — In silico analysis and locations of the target regions for the detection of Leptospira spp. for the primers and probe described by Smythe et al. (2002). Primers are denoted as arrow and probe as a green line, mismatches found in the sequences were denoted with red lines and the nucleotides are also shown. [file Image_4.PNG]
