# Supplementary material for: A Validated Multiplex Real-Time PCR Assay for the Diagnosis of Infectious Leptospira spp.: A Novel Assay for the Detection and Differentiation of Strains From Both Pathogenic Groups I and II
Source: Front Microbiol. 2020 Mar 20;11:457. doi: 10.3389/fmicb.2020.00457 (PMC7100377; doi:10.3389/fmicb.2020.00457)
Supplement: TABLE S3 — Sensitivity and specificity from Bayesian Latent class analysis. [file Table_3.docx]

**Table S3.** Sensitivity and specificity from Bayesian Latent class analysis**^a^**.

|  | **Test A (97.5%)** | **Test B (97.5%)** |
| --- | --- | --- |
| **Dse** | 96.45 | 96.66 |
| **DSp** | 95.18 | 96.68 |

**^a^**^:^ The model described by Li and Liu (2019) and available at Supplementary material Text1 was used. Dse: diagnostic sensitivity, Dsp: diagnostic specificity, Test A: qPCR (Smythe et al. 2002), Test B: qPCR multiplex presented here.
