# Supplementary material for: A Validated Multiplex Real-Time PCR Assay for the Diagnosis of Infectious Leptospira spp.: A Novel Assay for the Detection and Differentiation of Strains From Both Pathogenic Groups I and II
Source: Front Microbiol. 2020 Mar 20;11:457. doi: 10.3389/fmicb.2020.00457 (PMC7100377; doi:10.3389/fmicb.2020.00457)
Supplement: Supplementary file 8 [file Data_Sheet_1.docx]

**Supplementary Material TextS1**

model{

#Likelihood

x[1:4] ~ dmulti(p[1:4], n)

p[1] <- pi*((1-Se1)*(1-Se2)+covDp) + (1-pi)*(Sp1*Sp2+covDn)

p[2] <- pi*((1-Se1)*Se2-covDp) + (1-pi)*(Sp1*(1-Sp2)-covDn)

p[3] <- pi*(Se1*(1-Se2)-covDp) + (1-pi)*((1-Sp1)*Sp2-covDn)

p[4] <- pi*(Se1*Se2+covDp) + (1-pi)*((1-Sp1)*(1-Sp2)+covDn)

ls <- 0

us <- min(Se1,Se2) - Se1*Se2

lc <- 0

uc <- min(Sp1,Sp2) - Sp1*Sp2

covDn ~ dunif(lc, uc)

covDp ~ dunif(ls, us)

rhoD <- covDp / sqrt(Se1*(1-Se1)*Se2*(1-Se2))

rhoDc <- covDn / sqrt(Sp1*(1-Sp1)*Sp2*(1-Sp2))

#Prior

pi ~ dbeta(1,1)

Se1 ~ dbeta(1,1)

Sp1 ~ dbeta(1,1)

Se2 ~ dbeta(1,1)

Sp2 ~ dbeta(1,1)

}

#end BUGS model specification

#Data

list(x= c(560, 41, 22, 61), n = 684)

#Initial value

list(pi=0.51,Se1=0.50, Sp1=0.50, Se2=0.49, Sp2=0.51)
