# Supplementary material for: A Validated Multiplex Real-Time PCR Assay for the Diagnosis of Infectious Leptospira spp.: A Novel Assay for the Detection and Differentiation of Strains From Both Pathogenic Groups I and II
Source: Front Microbiol. 2020 Mar 20;11:457. doi: 10.3389/fmicb.2020.00457 (PMC7100377; doi:10.3389/fmicb.2020.00457)
Supplement: Supplementary file 9 [file Data_Sheet_2.docx]

**Supplementary information TextS2**

**Sequences obtained**

**Fig.5Bα:**

**>L pomona INFE Fwd/Rev consensus (172 nt)**

CGTGGGTAATCTTCCTCTGAGTCTGGGATAACTTTCCGAAAGGGAAGCTAATACTGGATGGTCCCGAGAGATCACAAGATTTTTCGGGTAAAGATTTATTGCTCGGAGATGAGCCCGCGTCCGATTAGCTAGTTGGTGAGGT

AAAGGCTCACCAAGGCGACGATCGTAGCCG

**Fig.5Bκ:**

**>L pomona Path Fwd/Rev consensus (153 nt)**

AGTTGGGCACTCGTAAGGAACTGCCGGTGACAAACCGGAGGAAGGCGGGGATGACGTCAAATCCTCATGGCCTTTATGTCTAGGGCAACACACGTGCTACAATGGCCGGTACAAAGGGTAGCCAACTCGCGAGGGGGAGCTAATCTCAAAAAA

**Fig.5Bδ:**

**>L broomii INFE LPath Fwd/Rev consensus (207 nt)**

GTGGGTAATCTTCCTCTGAGTCTGGGATAACTTTCCGAAAGGAAAGCTAATACCGGATAGTCCTACTGGATCACAGGATCTGATAGGTAAAGATTTATTGCTTGGAGATGAGCCCGCGGCCGATTAGCTAGTTGGTGAGGTAATGGCTCACCAAGGCGACGATCGGTAGCCGGCCTGAGAGGGTGTTCGGCCACAATGGAACTGAGA

**Fig.5Bσ:**

**>L broomii INT Fwd/Rev consensus (169 nt)**

GAGCGCAACCCCTATCGTATGTTGCTACCATTAAGTTGGGCACTCGTACGAAACTGCCGGTGACAAACCGGAGGAAGGCGGGGATGACGTCAAATCCTCATGGCCTTTATGTCCAGGGCCACACACGTGCTACAATGGCCGATACAGAGGGTCGCCAACTCGCAAGAGG

**Fig.5Bτ:**

**>L perolatii INT Fwd/Rev consensus (168 nt)**

GGAGCGCAACCCCTATCGTATGTTGCTACCATTAAGTTGGGCACTCGTACGAAACTGCCGGTGACAAACCGGAGGAAGGCGGGGATGACGTCAAATCCTCATGGCCTTTATGTCCAGGGCCACACACGTGCTACAATGGCCGATACAGAGGGTCGCCAACTCGCAAGA

**Sequence from sample:**

**>Query**

**GATTAGCTAGTTGGTGAGATAACTGCCCACCAAGGCGACGATCAGTAGCCGACCTGAGAGGGTG (Similarity to *L. biflexa* and *L. meyeri*)**
